# Optical properties of ZnMn_1.5_Al_0.5_O_4_ spinel nanoparticles: direct band gap, low Urbach energy, and estimated nonlinear optical response

**DOI:** 10.1039/d6ra06061g

**Published:** 2026-09-01

**Authors:** B. Abdellaoui, Mohamed Amghar, Mohsen Elain Hajlaoui, M. Bejar, B. F. O. Costa, E. Dhahri

**Affiliations:** a Laboratoire de Physique Appliquée, Faculté des Sciences, Université de Sfax 3000 Sfax Tunisia boutheinaabdelloui@gmail.com +216 96 162 555; b Faculté des Sciences de Monastir, Université de Monastir Tunisia; c University of Coimbra, CFisUC, Physics Department P-3004-516 Coimbra Portugal

## Abstract

In this work, the single-phase spinel oxide ZnMn_1.5_Al_0.5_O_4_(ZMAO) was successfully synthesized *via* the sol–gel auto-combustion method. X-ray diffraction analysis confirmed that the sample crystallizes in a tetragonal spinel structure with the *I*4_1_/*amd* space group. The optical properties, based on UV-visible-NIR reflectance measurements, reveal the semiconducting behavior of the ZMAO sample, with a direct optical band gap of approximately *E*_g_ = 2.160 ± 0.017 eV and a remarkably low Urbach energy (*E*_U_ = 0.376 ± 0.006 eV). The analysis of the optical constants of ZMAO reveals a refractive index of *n*_0_ ≈ 2.05 and a very low extinction coefficient of *k* = 1.8 × 10^−5^, indicating low optical absorption and promising potential for optical and optoelectronic applications. Using the Wemple–DiDomenico single effective oscillator model, the dispersion parameters *E*_0_ = 5.150 eV and *E*_d_ = 11.360 eV were determined, confirming the dispersive behavior of the material. In addition, the low dielectric losses (tan *δ* = 2.1 × 10^−5^) at high frequencies indicate a stable electrical response, further supporting the potential of ZMAO for electronic and functional devices. The third-order nonlinear optical susceptibility χ^(3)^ = 2.264 × 10^−21^ m^2^ V^−2^ and the nonlinear refractive index *n*_2_ = 2.662 × 10^−20^ m^2^·V^−2^ confirm the strong nonlinear optical response of ZMAO, suggesting its potential for applications in various nonlinear optical devices such as optical switches, modulators, and fiber-optic communication systems. The photoluminescence spectrum was deconvoluted into five emission bands. The 571 nm emission is attributed to near-band-edge recombination, whereas the 415–520 nm emissions are associated with localized electronic states, Mn-related states, and defects. The green emission around 520 nm is particularly linked to defect-related luminescence. Finally, CIE chromaticity analysis confirms that ZMAO is a promising material for optical applications, particularly as a phosphor for LEDs and solid-state lighting.

## Abstract 2

Photoluminescence spectra of the synthesized material ZMAO under 325 nm excitation, showing emission peaks at 415, 441, 480, and 520 nm.

## Graphical abstract

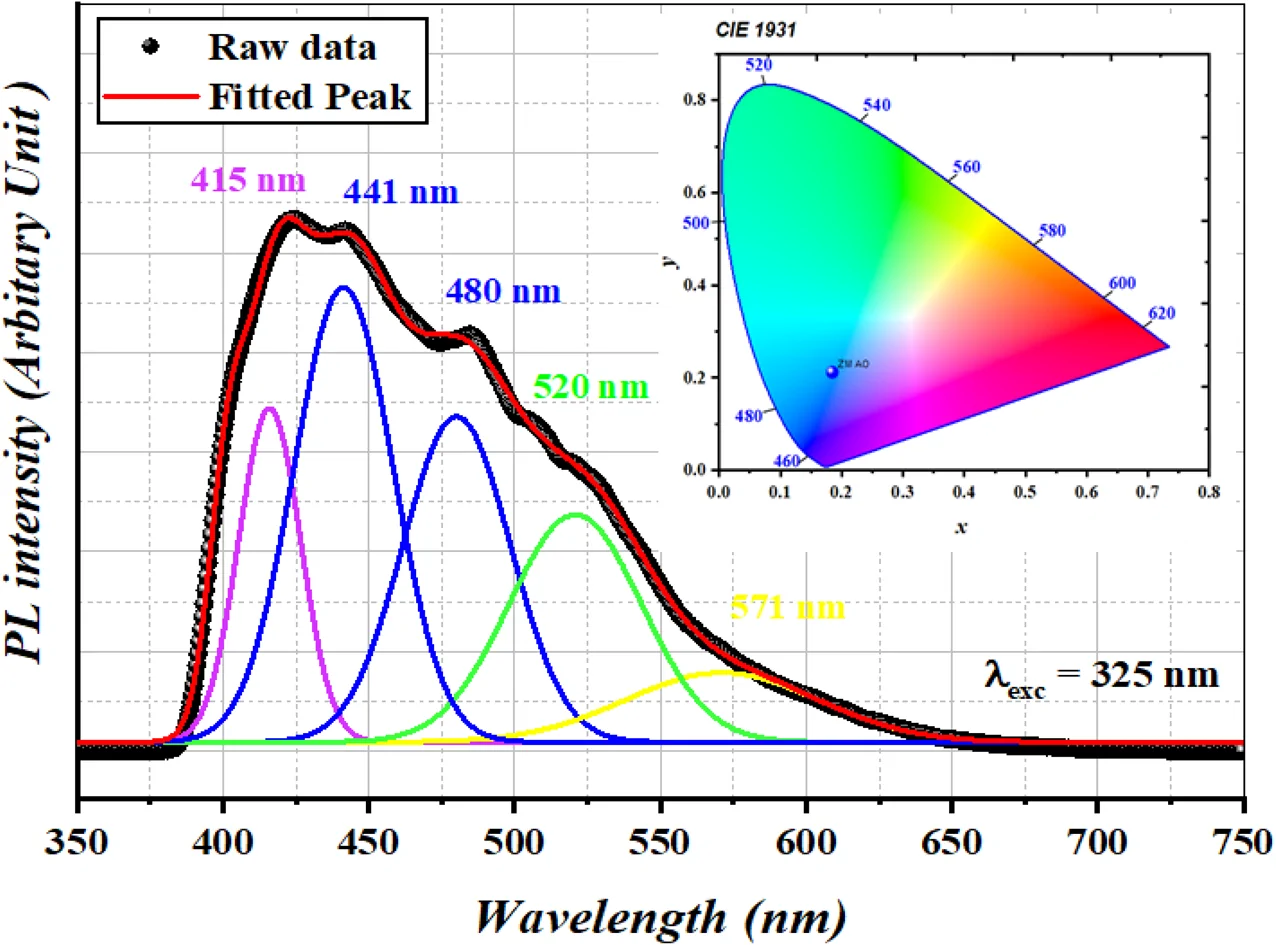

## 1 Introduction

In recent years, rapid technological progress has significantly stimulated research on miniaturized smart devices as well as high-performance and environmentally friendly energy systems. This has led to an increasing demand for lead-free multifunctional materials with tunable properties. In this context, semiconductor nanoparticles represent a strategic class of advanced materials due to their remarkable physicochemical properties. These properties make them highly suitable for a wide range of applications, including humidity sensors, devices operating under extreme conditions, heterogeneous catalysis, and integrated electronic systems.^1–4^ Furthermore, oxide and mixed-oxide ceramics have attracted considerable interest owing to their low synthesis temperatures, excellent chemical stability, and rich functional properties.^5^ Among them, transition-metal spinel oxides constitute a particularly promising family of materials, widely studied for their high thermal stability, good mechanical strength, tunable magnetic behavior, and strong catalytic activity.^6^ Moreover, spinel-structured manganates AMn_2_O_4_ have attracted considerable scientific interest due to their remarkable physicochemical properties.^7,8^ These materials are characterized by excellent structural stability, high chemical and thermal resistance, as well as tunable electrical and magnetic properties, which are strongly influenced by synthesis conditions and microstructural features.^9,10^ Over the past decade, manganese-based spinel materials have gained increasing attention owing to their strong potential for multifunctional applications.^11–13^ Spinel oxides have been widely investigated for various applications, including permanent magnets, microwave absorption devices, optoelectronic components, photovoltaic cells, and potential antimicrobial applications.^14–17^ In addition, these materials are also used as pigments for selective coatings in concentrated solar energy systems and in magnetic switching devices.^18,19^ In particular, recent studies have demonstrated that Zn- and Mn-based nanomaterials exhibit tunable optical absorption, band-gap energies, defect-related luminescence, and photoresponse, depending strongly on their composition, crystallinity, morphology, and defect concentration. For example, Senapati and Naik reported ratiometric optical temperature sensing based on greenish-white-emitting ZnO nanosheets, demonstrating the relevance of Zn-based nanostructures for optical sensing applications.^20^ Priyadarshini *et al.* showed that Zn incorporation into CuInSe_2_ nanosheets can modify the optical and dielectric characteristics, including the optical absorption and band-gap behavior, thereby improving their suitability for optoelectronic applications.^21^

More recently, Das *et al.* demonstrated that composition-dependent MnSe_1+*x*_T_1−*x*_ nanocomposites exhibit tunable optical band gaps, refractive indices, and measurable photodetection performance, highlighting the important role of Mn-based materials in optoelectronic devices.^22^ In particular, zinc manganite (ZnMn_2_O_4_, ZMO) is a promising semiconductor with a relatively narrow band gap (∼1.68 eV), good chemical stability, and remarkable redox activity.^23^ ZMO crystallizes in a tetragonal spinel structure with *I*4_1_/*amd* symmetry, in which Zn^2+^ ions preferentially occupy the tetrahedral sites, while Mn^3+^ ions are mainly located at the octahedral sites.^24^ The presence of multiple manganese oxidation states, together with structural defects, particularly oxygen vacancies, promotes charge-transport mechanisms and relaxation processes in this material.^25^ An important characteristic of ZnMn_2_O_4_ is the presence of Mn^3+^ ions, which are subject to the Jahn–Teller effect, leading to distortions of the MnO_6_ octahedra and consequently modifying the Mn–O bond lengths.^26^ These structural distortions can significantly affect the electronic structure, magnetic interactions, and charge-carrier transport mechanisms.

Numerous studies have shown that the incorporation of various heteroatoms (such as Ag^+^, Na^+^, K^+^, Ca^2+^, and Mg^2+^) can modify the electronic structure of electrode materials, thereby contributing to the stabilization of manganese-based cathodes.^27^ Among these dopants, Al^3+^ is particularly distinguished due to its high electronegativity and its strong interactions with the crystal lattice. Through the formation of stable Al–O bonds with oxygen atoms, Al^3+^ significantly enhances the structural stability of manganese-based cathodes.^28^ Furthermore, the close ionic radius of Al^3+^ to that of Mn^3+^ promotes the formation of new molecular orbitals, leading to a reduction in the band gap and an improvement in the electrical conductivity of manganese-based materials.^29^

The optical properties of ZnMn_2_O_4_ can be effectively tuned through cationic substitution, with their evolution depending strongly on the nature and concentration of the dopant. In this regard, ZnMn_2−*x*_V_*x*_O_4_ nanoparticles (0.0 ≤ *x* ≤ 0.4) exhibit an increase in the optical band-gap energy with increasing V^3+^ content, whereas ZnMn_2−*x*_Bi_*x*_O_4_ (0.0 ≤ *x* ≤ 0.2) shows enhanced absorption in the UV-visible region and a non-monotonic evolution of the optical band gap.^30,31^ Along the same lines, the incorporation of Fe^3+^ or Cr^3+^ into ZnMn_2−*x*_Fe_*x*_O_4_ and ZnMn_2−*x*_Cr_*x*_O_4_ (0.0 ≤ *x* ≤ 0.2) modifies the absorption in the visible region and enhances the light sensitivity.^32^

Among these different substitution strategies, the incorporation of Al^3+^ into ZnMn_2−*x*_Al_*x*_O_4_ (0.0 ≤ *x* ≤ 0.1) has been shown to modify the absorption characteristics and decrease the optical band gap with increasing Al content.^33^ This result suggests that the effect of Al^3+^ substitution on ZnMn_2_O_4_ warrants further investigation over a wider compositional range. Accordingly, a series of ZnMn_2−*x*_Al_*x*_O_4_ samples was systematically synthesized and characterized to assess the evolution of their structural, microstructural, and optical properties. Among the investigated compositions, ZnMn_1.5_Al_0.5_O_4_(*x* = 0.5) exhibited the most favorable overall response, with pronounced modifications in its structural and electronic environments.

In this context, Al^3+^-doped ZnMn_2_O_4_ nanoparticles with the chemical composition ZnMn_1.5_Al_0.5_O_4_ were successfully synthesized *via* the sol–gel auto-combustion method. The structural and morphological characteristics of the obtained samples were examined using X-ray diffraction (XRD) and scanning electron microscopy (SEM), the optical behavior was examined using UV-visible diffuse reflectance spectroscopy (UV-Vis DRS) and photoluminescence (PL) measurements. This study aims to explore the suitability of the synthesized nanomaterial for potential applications in advanced electronic devices. To the best of our knowledge, this is the first experimental study simultaneously reporting the linear and nonlinear optical constants (χ^1^, χ^2^, χ^3^, *n*_2_), the Wemple–DiDomenico dispersion parameters (*E*_0_, *E*_d_), the band edge positions (*E*^0^_CB_,*E*^0^_VB_), the photoluminescence deconvolution, and the complex optical modulus of Al^3+^-doped ZnMn_2_O_4_ nanoparticles with the specific stoichiometry ZnMn_1.5_Al_0.5_O_4_ synthesized by the sol–gel auto-combustion route. While prior studies on ZnMn_2_O_4_ have focused primarily on magnetic, electrochemical, or gas-sensing properties, the present work provides a comprehensive optical and photonic characterization of the Al-doped variant, revealing its strong potential for future optoelectronics and nonlinear photonic devices.

## 2 Experimental

The ZnMn_1.5_Al_0.5_O_4_ compound was synthesized using the sol–gel auto-combustion method.^26^ This synthesis route is widely used and highly attractive for the preparation of nanostructured materials due to its several advantages, including low processing temperature, homogeneous distribution of reactants, and the ability to produce materials with high crystallinity, narrow particle size distribution, and uniform morphology.^27^ Stoichiometric amounts of Zn(NO_3_)_2_·6H_2_O, MnSO_4_·H_2_O, and Al(NO_3_)_3_·9H_2_O were used as precursor salts to provide the required metal cations for the formation of the spinel structure. Each precursor was dissolved separately in 100 ml of distilled water. The resulting solutions were then homogenized under constant stirring to obtain a homogeneous solution, which was then heated to 80 °C to form a viscous gel. The obtained gel was further heated on a hot plate at 180 °C to promote its transformation into a powder. The resulting powder was ground and calcined in air at 400 °C for 24 h to obtain a dry porous mass. To remove residual organic components, the powder was subsequently annealed at 600 °C for 24 h. It was then reground and calcined at 700 °C for 24 h to improve crystallinity. Finally, the powder was pressed into pellets of 8 mm diameter and sintered at 800 °C for 24 h to enhance crystallization. The X-ray diffraction (XRD) patterns were recorded using a D8 Advance diffractometer equipped with a CuKα radiation source (*λ* = 1.5406 Å). The measurements were performed over the (2theta) range of 10–100°, with a step size of 0.02°. The morphology of the synthesized sample was investigated by scanning electron microscopy (SEM, SU5000) at an accelerating voltage of 15.0 kV, equipped with an EDX detector. The diffuse UV-visible reflectance spectra were recorded using a SHIMADZU UV-3101 PC spectrophotometer over the wavelength range of 200–1000 nm. The photoluminescence (PL) spectra were recorded at room temperature using a high-resolution Avantes CCD detector, under excitation at *λ*_ex_ = 325 nm provided by a tunable Ti laser (Spectra-Physics 3900S).

## 3 Results and discussion

### 3.1 Structural analysis

**Fig. 1:**
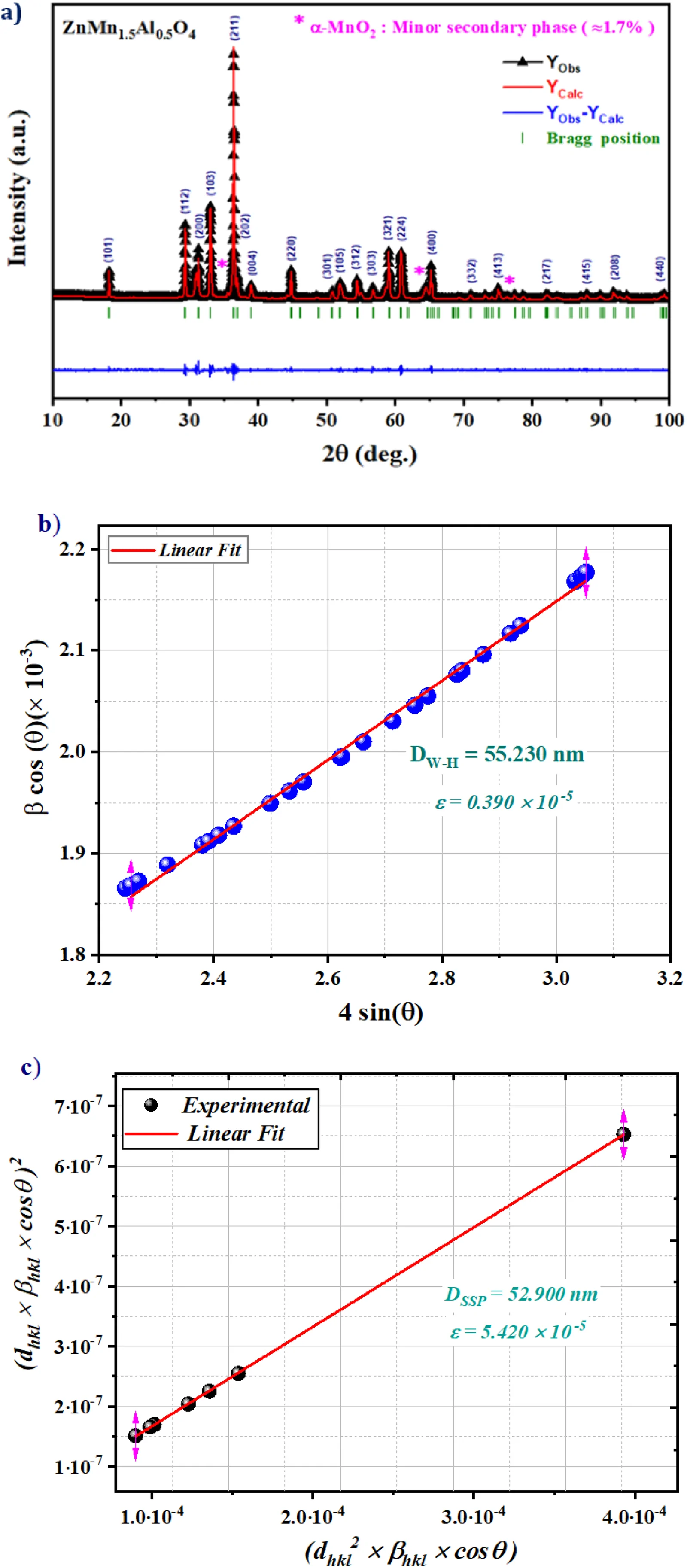
(a) Rietveld refinement pattern, (b) Williamson–Hall (W–H) plot, and (c) average crystallite size estimated using the strain–size plot (SSP) method for the ZnMn_1.5_Al_0.5_O_4_ sample.

The X-ray diffraction (XRD) analysis confirmed that the ZnMn_1.5_Al_0.5_O_4_ ceramic adopts a tetragonal spinel structure belonging to the *I*4_1_/*amd* space group, as illustrated in Fig. 1(a). The XRD patterns were used to identify the crystalline phases present in the ZnMn_1.5_Al_0.5_O_4_ sample. The observed diffraction peaks were clearly indexed to the crystallographic planes (101), (112), (200), (103), (211), (202), (004), (220), (213), (301), (105), (312), (303), (321), (224), and (116), confirming the formation of a well-crystallized tetragonal spinel structure. However, a very weak additional peak appears in the diffraction pattern, suggesting the presence of a minor secondary phase identified as α-MnO_2_, formed during the synthesis process. The fraction of this impurity phase is estimated to be less than 3%, indicating that the sample remains predominantly single-phase and that the main crystal structure is not significantly affected.

Furthermore, the low value of the goodness-of-fit parameter *χ*^2^ = 1.7 reveals an excellent agreement between the experimental and calculated diffraction patterns, thus demonstrating the high quality of the Rietveld refinement. These results ensure an accurate interpretation of the XRD data and confirm the absence of other significant secondary phases. The results of the Rietveld refinement are illustrated in Table 1.

**Table 1:** Summary of structural parameters obtained from XRD pattern of the ZnMn_1.5_Al_0.5_O_4_ sample

ZnMn_1.5_Al_0.5_O_4_	
*a* (Å)	5.72_0_
*c* (Å)	9.25_9_
*V* (Å^3^)	302.97_5_
*χ* ^2^	1.7
*ρ*_X-ray_ (g cm^−3^)	4.93
*ρ*_exp_ (g cm^−3^)	4.67
*P* (%)	5.85
*D*_W−H_ (nm)	55.230
*ε* (×10^−5^)	0.390
*δ* (×10^−3^)	3.280
*D*_SSP_ (nm)	52.900
*ε* (×10^−5^)	5.420

The X-ray density (*ρ*_X-ray_) was calculated by assuming that the unit cell of the spinel structure contains four formula units (*Z* = 4), according to the following relation:^34^1
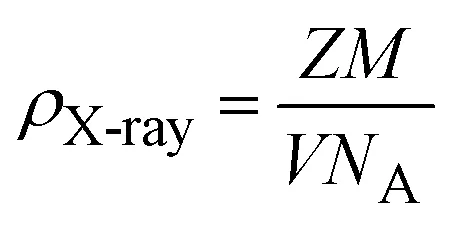
where *Z* = 4 represents the number of formula units per unit cell, *M* is the molar mass of the compound, *V* is the unit cell volume (Table 1), and *N*_A_ = 6.022 × 10^23^ mol^−1^ is Avogadro's number. The X-ray density *ρ*_X-ray_ value indicated in Table 1 was compared to the experimental density (*ρ*_exp_) value determined using the following relation:^34^2
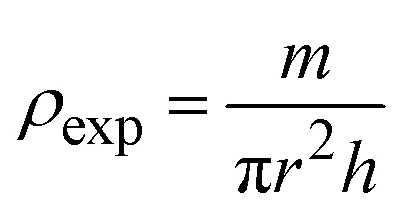
where *h* = 1.150 mm, *r* = 4 mm, and *m* = 0.270 g represent the thickness, radius, and mass of the pellet, respectively. Table 1 indicates that the *ρ*_exp_ value closely matches the *ρ*_X-ray_ one. Once the *ρ*_X-ray_ and *ρ*_exp_ values were determined, the porosity (*P*) was calculated using the following relation:^35^3
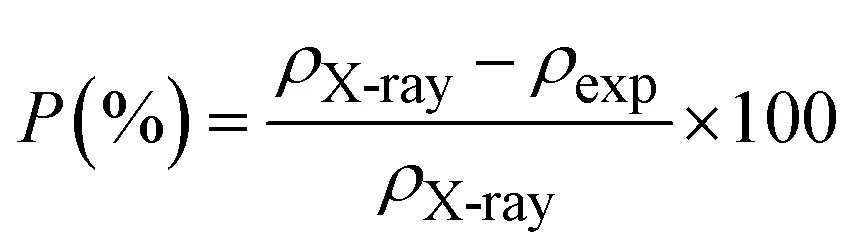


The determined low porosity value *P* = 5.85% (Table 1) indicates a dense microstructure characterized by good grain packing, homogeneous particle distribution, and strong intergranular cohesion.

The refined lattice parameters of ZMAO *a* = 5.720 Å and *c* = 9.259 Å, with a unit-cell volume of 302.975 Å^3^, are consistent with those reported for tetragonal ZnMn_2_O_4_ spinels (*a* = 5.715 Å, *c* = 9.252 Å, and *V* = 302 Å^3^).^36^ Al substitution causes only slight changes in the lattice parameters while preserving the tetragonal *I*4_1_/*amd* structure,^37^ suggesting local lattice relaxation and cation redistribution. The theoretical and experimental densities of 4.93 and 4.67 g cm^−3^, respectively, indicate low residual porosity and satisfactory densification of the sintered ceramic.

The crystallite size (*D*_W–H_) was estimated using the Williamson–Hall method based on the following equation:^38^4
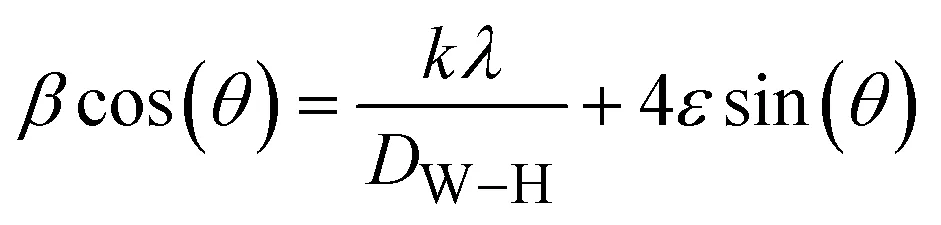
where *k* = 0.9, *λ* = 1.5406 Å (Cukα), *β* is the full width at half maximum (FWHM), *θ* is the Bragg angle, and *ε* is the microstrain.

The Williamson–Hall plot (Fig. 1(b)), obtained by plotting *β* cos(*θ*) *versus* 4sin(*θ*), allowed the simultaneous determination of the crystallite size (*D*_W–H_) and the microstrain (*ε*), yielding values of *D*_W–H_ = 55.230 nm and *ε* = 0.390 × 10^−5^, indicating a slight lattice expansion associated with internal stress effects.^38,39^ The positive value of the residual stress indicates the presence of tensile stress within the compound.^40^ The microstrain determined for our sample is *ε* = 0.390 × 10^−5^, which is significantly lower than the values reported in the literature, namely 6.09 × 10^−3^ and 8.97 × 10^−3^.^41^ This significant difference suggests that our material exhibits a low level of lattice microstrain, which may be associated with limited accumulation of local distortions and better accommodation of stress within the crystal structure.

Based on the determined *D*_W–H_ value, the dislocation density (*δ*) was calculated as:^38^5
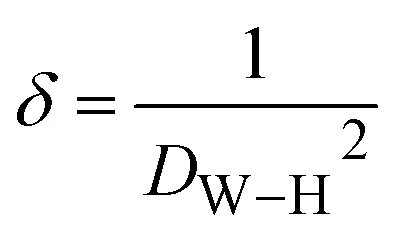


The obtained value is *δ* = 3.280 × 10^−3^, indicating a low density of crystallographic defects. This result confirms the good crystallinity of the ZnMn_1.5_Al_0.5_O_4_ compound, as well as a relatively ordered crystal structure with minimal lattice imperfections.

Moreover, to investigate the microstructural characteristics of the studied ZnMn_1.5_Al_0.5_O_4_ aluminate spinel material, the well-known size–strain plot (SSP) method was employed to estimate the average crystallite size. This approach is expressed by the following relation:^38^6
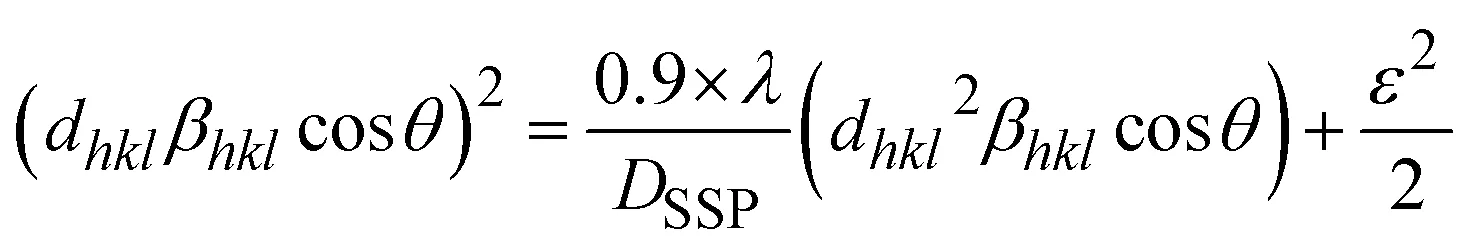
In Fig. 1(c), (*d*_*hkl*_*β*_*hkl*_ cos *θ*)^2^ was plotted against (*d*_*hkl*_^2^ *β*_*hkl*_ cos *θ*), for all diffraction peaks of the ZnMn_1.5_Al_0.5_O_4_ sample. The crystallite size (*D*_SSP_) was determined from the slope of the linear fit and found to be 52.900 nm, while the intercept yielded a microstrain value of *ε* = 5.420 × 10^−5^. The crystallite size obtained from the SSP method and Williamson–Hall (W–H) confirms the effectiveness of the sol–gel auto-combustion route for sample synthesis. Moreover, the diffraction peak broadening associated with lattice strain indicates the presence of noticeable crystalline distortions induced by Al incorporation.

### 3.2 Scanning electron microscopy (SEM) analysis

**Fig. 2:**
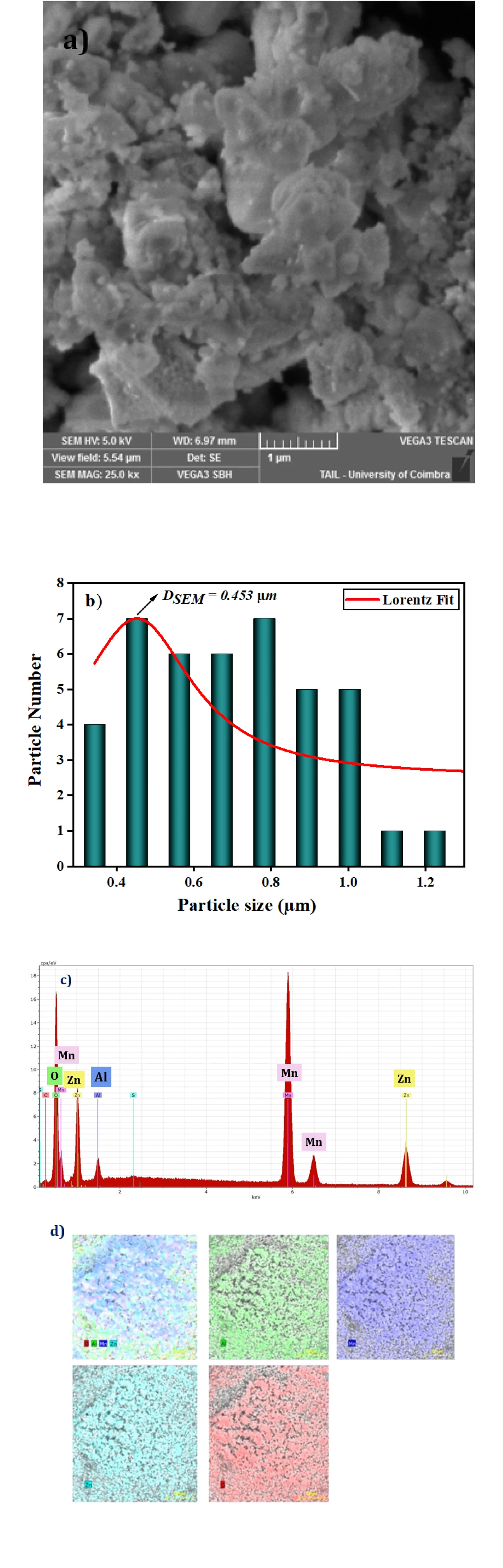
(a) SEM image, (b) particle size histogram based on a Lorentzian distribution, (c) EDS spectrum, (d) elemental mapping of the ZnMn_1.5_Al_0.5_O_4_ sample.

The surface morphology of the ZnMn_1.5_Al_0.5_O_4_ sample was studied by scanning electron microscopy (SEM), as illustrated in Fig. 2(a). The obtained micrographs reveal the formation of polycrystalline agglomerates composed of fine particles of irregular shapes and sizes. The grains exhibit a relatively heterogeneous particle size distribution, while the observed agglomerates appear to result from the assembly of primary nanoparticles. To quantify the particle size distribution, the SEM images were carefully processed using ImageJ software, resulting in the particle size histograms shown in Fig. 2(b). From these distributions, the mean size of the agglomerates was estimated to be approximately *D*_SEM_ = 0.453 µm. Furthermore, a comparison between the mean crystallite size determined by X-ray diffraction and the particle size obtained by SEM shows that the latter is larger. This difference suggests that each grain observed by SEM is made up of several crystallites, thus confirming the polycrystalline nature of the agglomerates formed during the synthesis process.

The EDS spectrum presented in Fig. 2(c) confirms the presence of Zn, Mn, Al, and O in the ZnMn_1.5_Al_0.5_O_4_ sample, in agreement with its nominal chemical composition. The characteristic Mn peaks exhibit relatively high intensity, which is consistent with its higher content in the investigated composition. A weak carbon (C) signal is also observed at low energy, which can be attributed to the conductive substrate used during the analysis. In addition, a weak sulfur (S) peak is detected, which may originate from residual traces associated with the precursors used during the synthesis. Furthermore, the elemental mapping shown in Fig. 2(d) reveals a relatively homogeneous spatial distribution of Zn, Mn, Al, and O throughout the analyzed region.

### 3.3 UV-visible/NIR spectroscopy

The UV-visible spectroscopy is widely employed as a characterization technique across various scientific fields, photonics, solar cell technologies, and light-emitting devices. It is particularly well suited for quantitative analysis due to the linear relationship between absorbance and the concentration of absorbing species.

In molecular and inorganic materials, the absorption of ultraviolet or visible photons promotes electrons to higher energy states. Therefore, this technique serves as an effective tool for investigating the optical properties of semiconducting materials, both in fundamental research and technological applications. It is commonly used to study a broad range of materials, such as solid-state laser hosts, photovoltaic absorber materials, and materials designed for optical information processing and high-speed optical communications. In the present study, the optical properties of the spinel ZnMn_1.5_Al_2_O_4_ sample were analyzed based on reflectance measurements. These investigations provide valuable insights into the nature of optical transitions (direct or indirect), dispersive behavior, and the spectral evolution of key optical parameters such as the extinction coefficient (*k*) and refractive index (*n*). They also enable the evaluation of the material's band structure, helping to identify its potential applications.

#### 3.3.1 Reflectance study

**Fig. 3:**
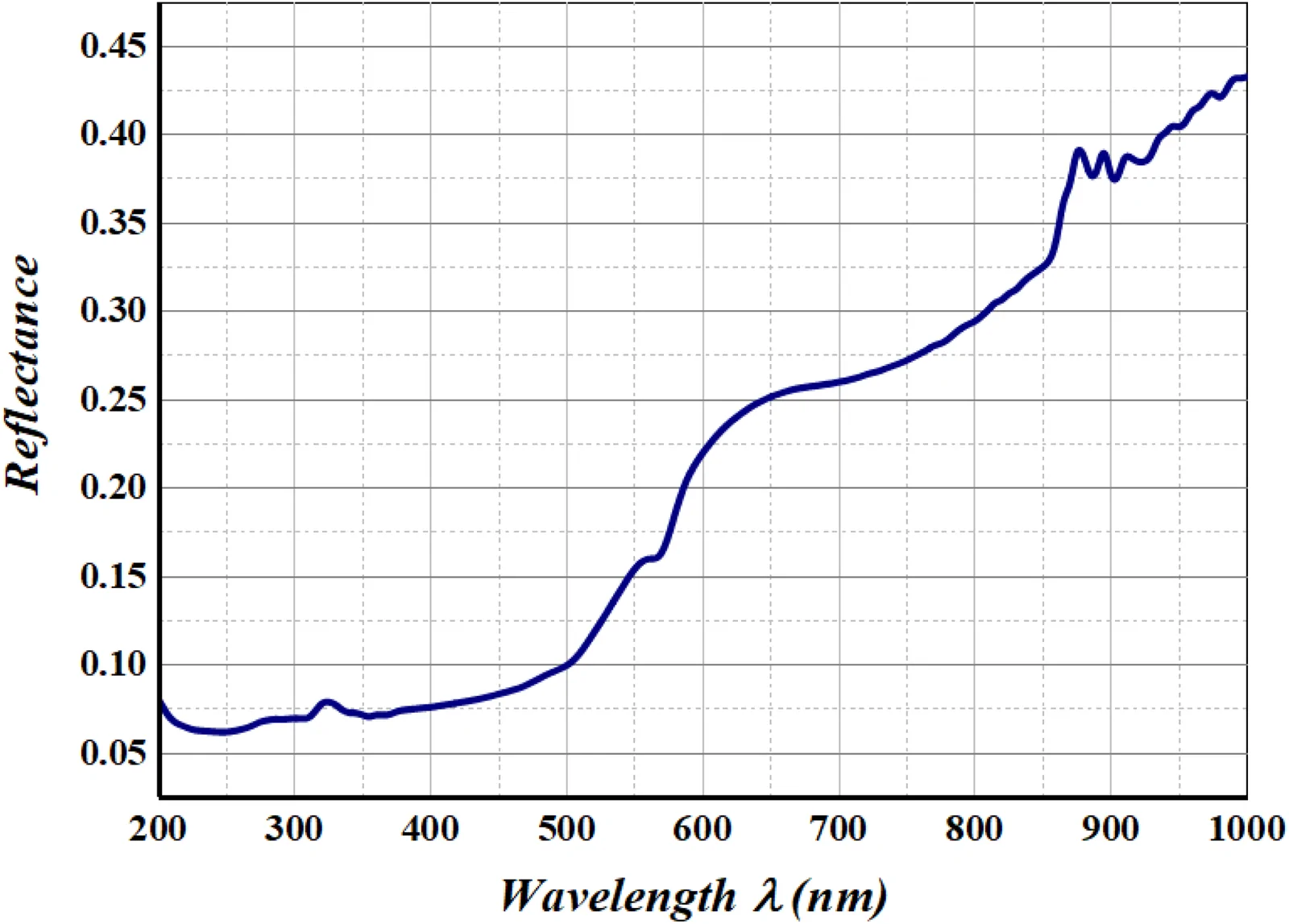
Reflectance optical spectrum for the ZnMn_1.5_Al_0.5_O_4_ sample.

UV-visible-NIR spectroscopy was used to investigate the spectral behavior of the optical reflectance of the ZMAO compound over the wavelength range from 200 to 1000 nm, as shown in Fig. 3. The obtained spectrum reveals a gradual increase in reflectance with increasing wavelength, indicating strong absorption in the UV-visible region and enhanced reflection in the near-infrared region. A pronounced variation is observed around 550–600 nm, corresponding to the optical absorption edge, which is associated with electronic transitions between the valence band and the conduction band, confirming the semiconducting nature of the material. Overall, these results highlight the potential interest of this compound for diverse applications in both the visible and near-infrared regions.

#### 3.3.2 Optical band gap energy

To better understand the optical behavior of the investigated material, it is essential to accurately determine the band gap energy (*E*_g_), which is one of the fundamental properties of semiconductor materials. A low *E*_g_ value facilitates the excitation of electrons from the valence band to the conduction band, whereas a high *E*_g_ value indicates a higher excitation energy and may favor greater transparency in the spectral range considered. In contrast, the fundamental gap is defined as the energy difference between the first electron affinity and the first ionization potential.^42^ Generally, the optical gap is smaller than the fundamental gap due to the electrostatic interaction between the electron and the hole, which remain bound in the excited state (exciton), unlike the fully ionized state where they are separated.^43^ The optical band gap energy (*E*_g_) of the manganese-based spinel compound ZMAO can be estimated using Tauc's relation, given by the following formula:^44^

7 (*F*(*R*) *hv*)^1/*n*^ = *A*(*hv* − *E*_g_)

where 
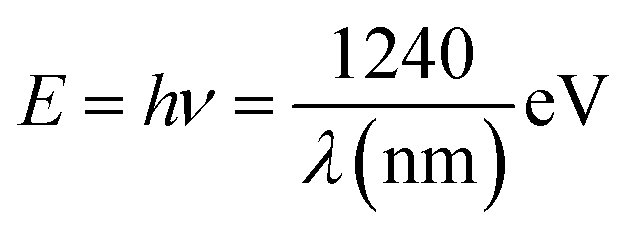
 denotes the incident photon energy and, *A* represents a material-dependent proportionality constant related to the probability of optical transitions and the electronic properties of the material, particularly its electronic structure, transition matrix elements, and density of electronic states. Its value primarily influences the slope of the Tauc plot and thus reflects the intrinsic characteristics of the optical absorption process.^45,46^

The parameter *n* is an exponent associated with the type of electronic transition, which depends on the semiconducting behavior of the material: it corresponds to a directly allowed transition (*n* = 1/2), an indirectly allowed transition (*n* = 2), or a forbidden transition. The function *F*(*R*) refers to the Kubelka–Munk function, which can be expressed in terms of diffuse reflectance data *R* as follows:^47^8
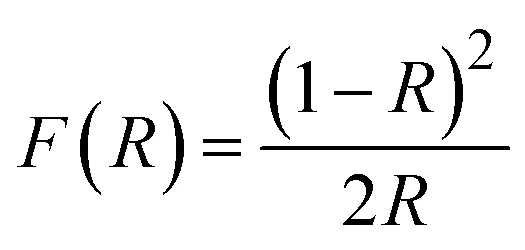


This function was converted into an equivalent linear absorption coefficient using the following relation:^48^9
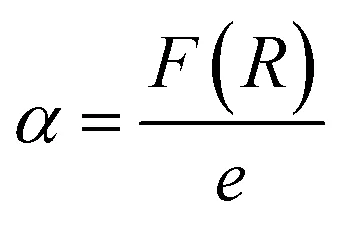
where (*e* = 1.15 mm) denotes the thickness of the sample.

The optical band gap energy (*E*_g_) is determined by extrapolating the linear region of (*F*(*R*)*hv*)^1/2^ (*n* = 2) and (*F*(*R*)*hv*)^2^ (*n* = 0.5) as a function of the incident photon energy (*hν*). The most appropriate band gap energy is obtained for *n* = 0.5. Thus, for a direct band gap semiconductor, Tauc's law can be expressed as follows:

10 (*F*(*R*)*hv*)^1/2^ = *A*(*hv* − *E*_g_)

**Fig. 4:**
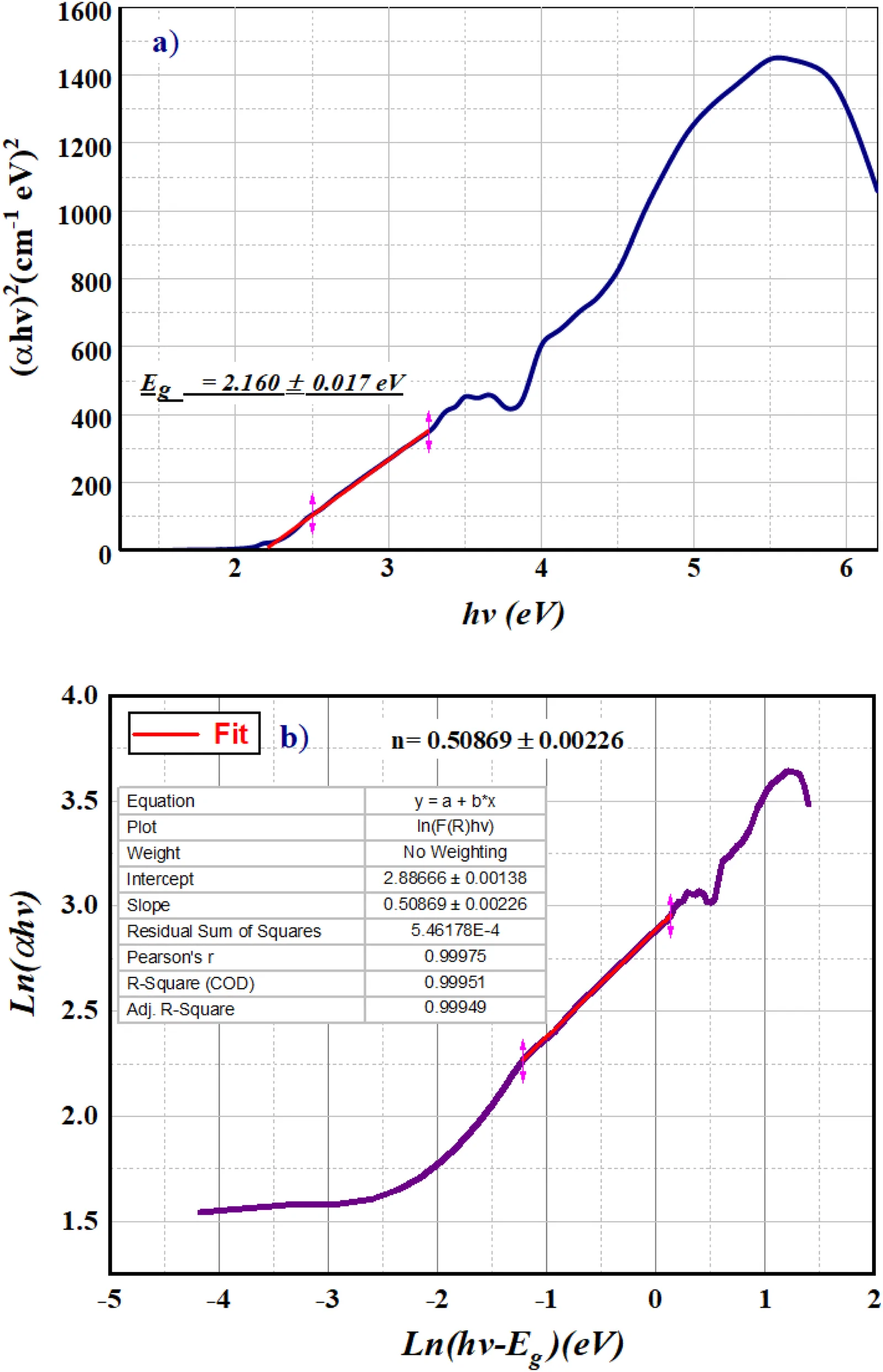
(a) The plot of (*αhν*)^2^*vs. hν* and (b) the optical transition mode for the ZnMn_1.5_Al_0.5_O_4_ sample.

The *E*_g_ value of the ZMAO sample, determined using Tauc's law as shown in Fig. 4(a), is estimated to be 2.160 eV. This value is lower than those reported for similar systems, including Amghar *et al.* (*E*_g_ = 4.000–4.300 eV),^49^ Zein K. Heiba *et al.* (*E*_g_ = 2.480–2.580 eV),^32^ and Cecilia Guillén *et al.* (*E*_g_ = 3.140–3.340 eV).^50^ This reduction in band gap energy can be attributed to cation redistribution within the spinel lattice, as well as structural disorder and defect states that introduce localized energy levels within the forbidden band gap, thereby facilitating electronic transitions. The obtained narrow band gap further confirms the suitability of the ZMAO material for optical applications, owing to its enhanced light absorption capability and improved charge carrier activation.

To verify the direct nature of the band gap transition, a mathematical approach can be employed:

11 ln(*F*(*R*)*hv*) = ln(*A*) + *n*ln(*hv* − *E*_g_)

Based on the spectral variation of ln(*F*(*R*)*hν*) as a function of ln(*hν* − *E*_g_), the power factor (*n*), which reflects the nature of the electronic transition, is obtained from the slope of the linear fitting shown in Fig. 4(b). Consequently, a linear relationship can be established as follows:

12 ln(*F*(*R*)*hv*) = 2.886 + 0.5 ln(*hv* − 2.160)

The direct band gap semiconducting behavior has been successfully confirmed. The experimental value of *n* is in good agreement with the theoretically predicted transition type. It should be emphasized that applications devices based on direct band gap materials offer a major advantage. In these materials, the energy released during electron–hole recombination is predominantly emitted in the form of light, with minimal involvement of phonons (quasi-particles associated with lattice vibrations). Thus, direct band gap materials are particularly attractive for optical and electronic applications, especially in detectors and sources of infrared radiation involved in interband transitions.^51^ The band gap width, as well as its dependence on the wave vector *k*, are fundamental parameters for understanding the behavior of semiconductors.^52^ To further analyze the band structure of the studied material, it is necessary to determine the positions of the conduction band edge (*E*^0^_CB_) and the valence band edge (*E*^0^_VB_), which can be calculated using the following formulas:^52^

13 *E*^0^_CB_ = *E*_e_ − *χ* + 0.5*E*_g_

**Fig. 5:**
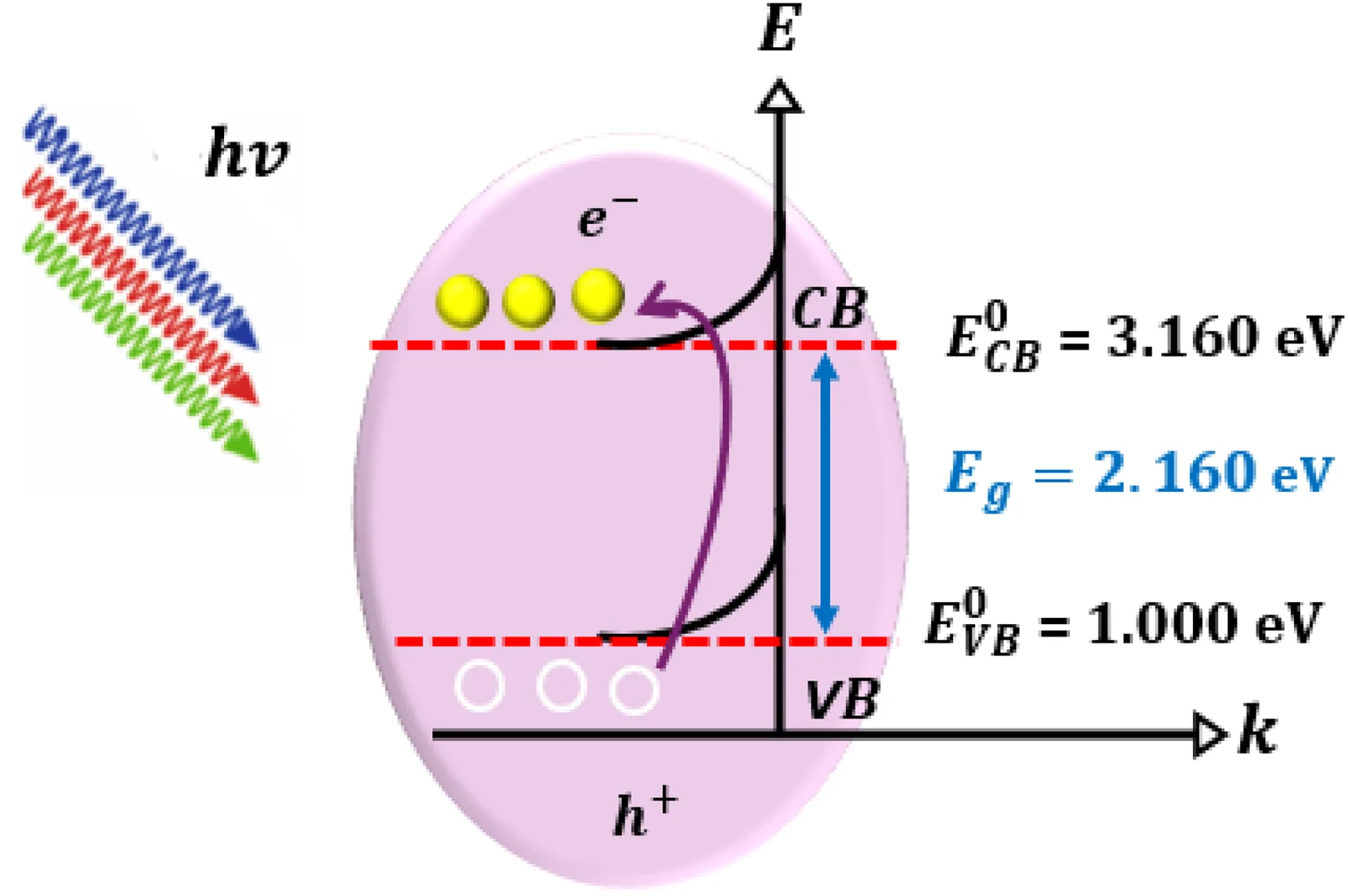
Band structure for the ZnMn_1.5_Al_0.5_O_4_ sample.

14 *E*^0^_VB_ = *E*^0^_CB_ − *E*_g_

where *E*_e_ denotes the energy of the free electron on the hydrogen scale (*E*_e_ ≅ 4.5 eV), and *χ* represents the electronegativity of the studied ZnMn_1.5_Al_0.5_O_4_ spinel manganese sample, which can be calculated as follows:^53^15
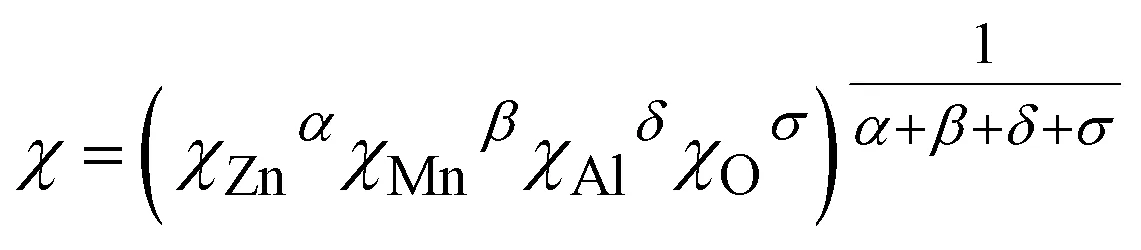
In this expression, *α* = 1, *β* = 1.5, *δ* = 0.5 and *σ* = 4 represent the atomic multiplicities of Zn, Mn, Al, and O, respectively. The electronegativity values used are *χ*_Zn_ = 1.65, *χ*_Mn_ = 1.55, *χ*_Al_ = 1.61 and *χ*_O_ = 3.44. Based on the previous expressions, the calculated values of χ, *E*^0^_CB_, and *E*^0^_VB_ were found to be 2.420, 3.160 eV and 1.000 eV, respectively. Consequently, a schematic representation of the band structure can be proposed, as illustrated in Fig. 5.

In summary, these results demonstrate that ZnMn_1.5_Al_0.5_O_4_ sample is a promising material for optical applications in the visible region.^54^ The band gap value (*E*_g_ = 2.160 eV) indicates absorption and emission within the visible range. Furthermore, the presence of a direct allowed transition enhances the radiative recombination efficiency.

#### 3.3.3 Urbach energy

The study of the Urbach energy, denoted as *E*_U_, is an important parameter for evaluating structural disorder, impurities, crystallographic defects, and the broadening of electronic transitions in semiconductor materials. A low *E*_U_ value generally indicates a relatively well-ordered material, whereas a high value indicates an increased degree of disorder and the presence of localized electronic states.

These imperfections generate absorption states located in the intermediate region between the valence band and the conduction band, known as the Urbach tail, which can influence the physical properties of the material and, consequently, its performance in applications.^55,56^ The determination of the Urbach energy *E*_U_ thus provides a better understanding of localized states extending into the band gap on both sides of the forbidden energy region.^57^ It also gives insight into the exponential decay of these states and is inversely related to their spatial extent. The Urbach energy can be expressed as a function of the incident photon energy as follows:^57^16
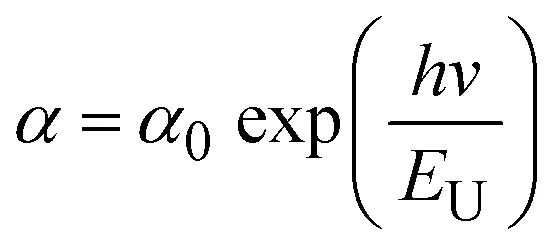
where *α* is optical absorption coefficient of the material (cm^−1^), and *α*_0_ pre-exponential constant.

By applying a Napierian logarithm to eqn (16), we obtain 57:17
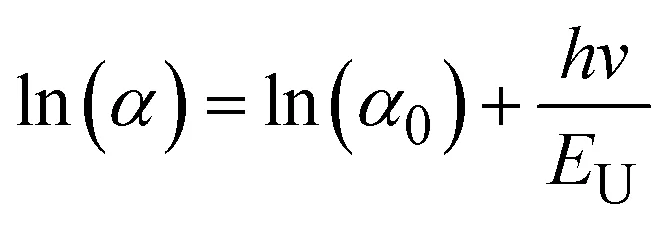


The Urbach energy (*E*_U_) was determined from the linear fitting of ln(*α*) *versus* photon energy (*hν*). The fitting was performed in the exponential absorption region, within the energy range of 2.2–2.5 eV.

This parameter is commonly used to evaluate the degree of structural disorder and the density of localized states within the band gap. A low *E*_U_ value indicates a reduced level of structural disorder and fewer defect states, whereas a higher value reflects an increased density of defects and disorder in the material.

**Fig. 6:**
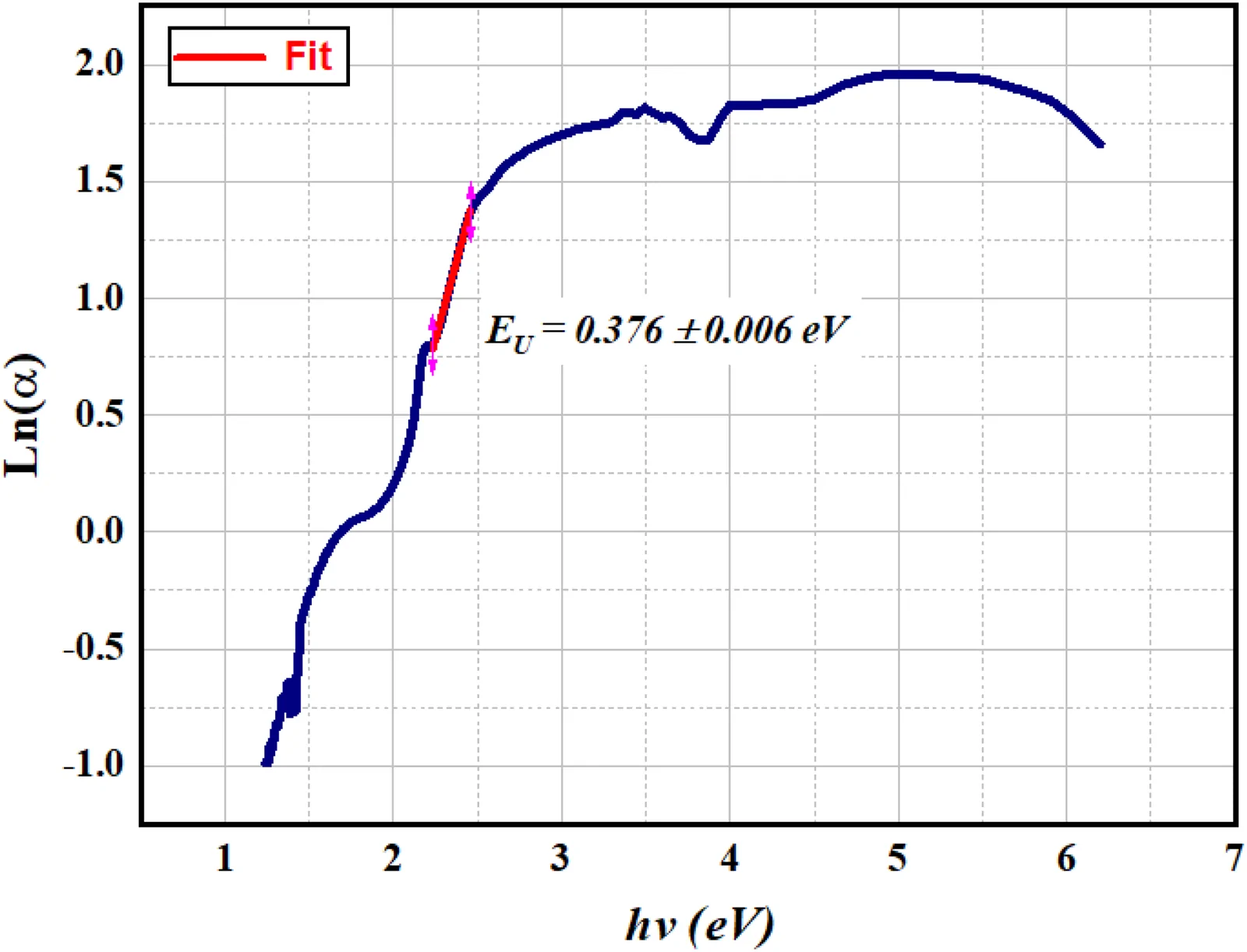
Relation between ln(*α*) and photon energy (*hν*) for the ZnMn_1.5_Al_0.5_O_4_ sample.

For the ZMAO sample investigated in this work, the Urbach energy was estimated to be *E*_U_ = 0.376 ± 0.006 eV (Fig. 6). For comparison, this value is significantly lower than those reported by A. Alqahtani *et al.* (*E*_U_ = 1.386–2.275 eV)^58^ and L. H. Omari *et al.* (*E*_U_ = 1.003–1.031 eV).^59^ According to Sasmita Giri *et al.*,^40^ variations in the Urbach energy are associated with changes in the density of localized defect states within the band gap. The relatively low *E*_U_ value obtained in the present study therefore suggests a lower degree of structural disorder and a reduced density of localized defect states, indicating improved structural and crystalline quality of the synthesized spinel.

Furthermore, interactions between electrons and phonons, as well as between electrons and excitons, lead to a broadening of the absorption edge. This effect is described by the steepness parameter, which can be expressed as a function of the incident photon energy according to the exponential Urbach law:^60^18
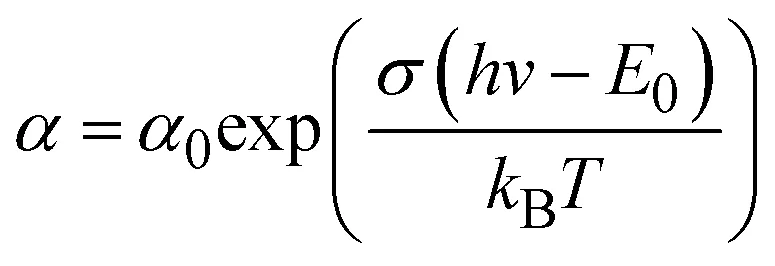
In this relation, *k*_B_ denotes the Boltzmann constant, *E*_0_ represents the energy of the lowest excitonic state at zero lattice temperature and *σ* is steepness parameter. According to the literature^61^*E*_0_ is equal to *E*_g_ in the case of direct band gap semiconductors, while it is expressed as *E*_g_ ± *E*_ph_ for indirect band gap semiconductors, where *E*_ph_ is the phonon energy.

By applying the natural logarithm to eqn (18), the following relation can be obtained:19
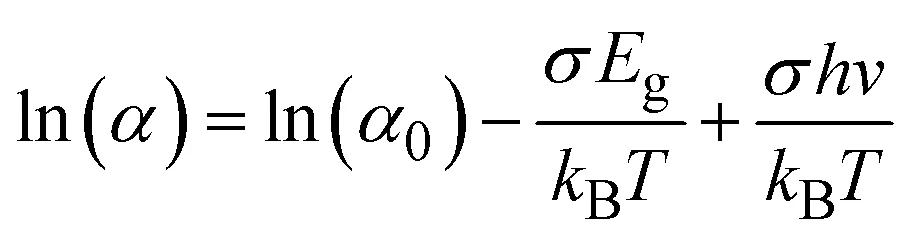
By comparing Eqs (18) and (19), we can deduce:^61^20
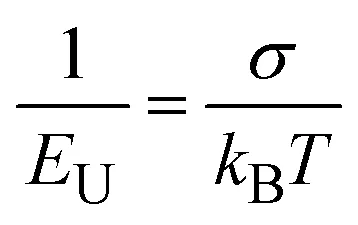


The steepness parameter (*σ*) provides information on how the absorption coefficient varies with the incident photon energy near the absorption onset.^62^ In this study, the value of *σ* was estimated to be 0.069.

Furthermore, the electron–phonon interaction energy *E*_e–ph_ can be related to this parameter through the following expression:^61^21
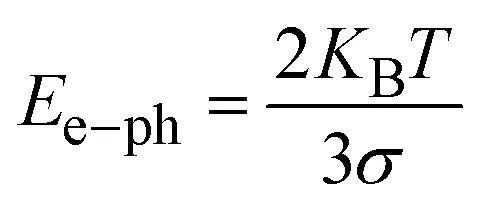


The calculated value of *E*_e–ph_ is approximately 0.251 eV. This parameter offers valuable insight into the electronic and thermal properties of semiconductors. A higher *E*_e–ph_ value indicates stronger electron–phonon interaction, reflecting an enhanced ability of the material to dissipate energy through lattice vibrations. This relatively low value (0.251 eV) suggests that the electron–phonon coupling in ZMAO is moderate, which is favorable for applications as it reduces non-radiative recombination losses.

#### 3.3.4 Linear and non-linear optical response

One of the fundamental optical properties of materials is the complex refractive index (*n̂*), defined by:^63^

22 *n̂* = *n* + *ik*

where *n* and *k* represent the real part (refractive index) and the imaginary part (extinction coefficient), respectively.

A high value of *n* indicates that light propagates more slowly through the material and generally reflects strong electronic polarization as well as significant interaction between the material and the electromagnetic field. Therefore, the refractive index is an essential parameter for optical and photonic applications, particularly in waveguides and nonlinear optical devices.^64^ Moreover, *k* is directly associated with optical losses, which are mainly related to intrinsic absorption and electronic transitions. Thus, the spectral evolution of *k* provides important information about the absorption mechanisms and electronic transitions responsible for the optical properties of the material.^65^

These two quantities can be determined using the following Kramers–Kronig relations:^66^23
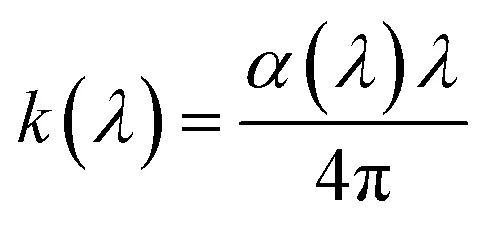
24
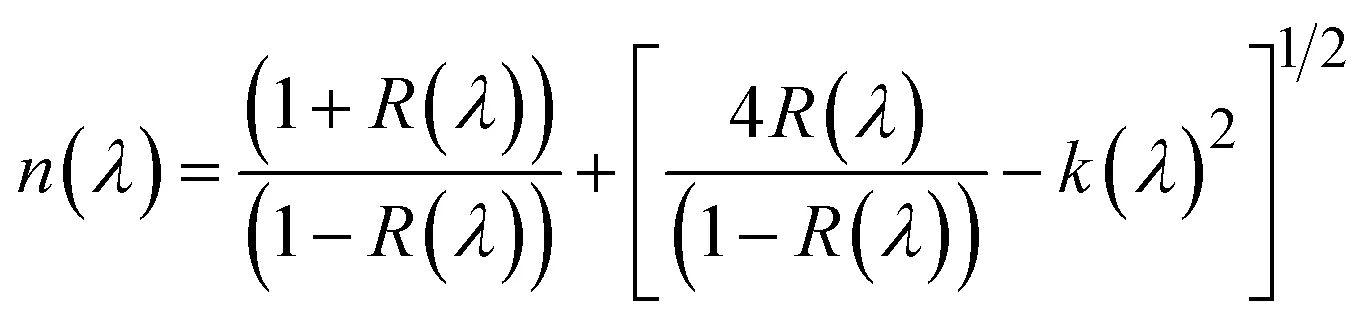


**Fig. 7:**
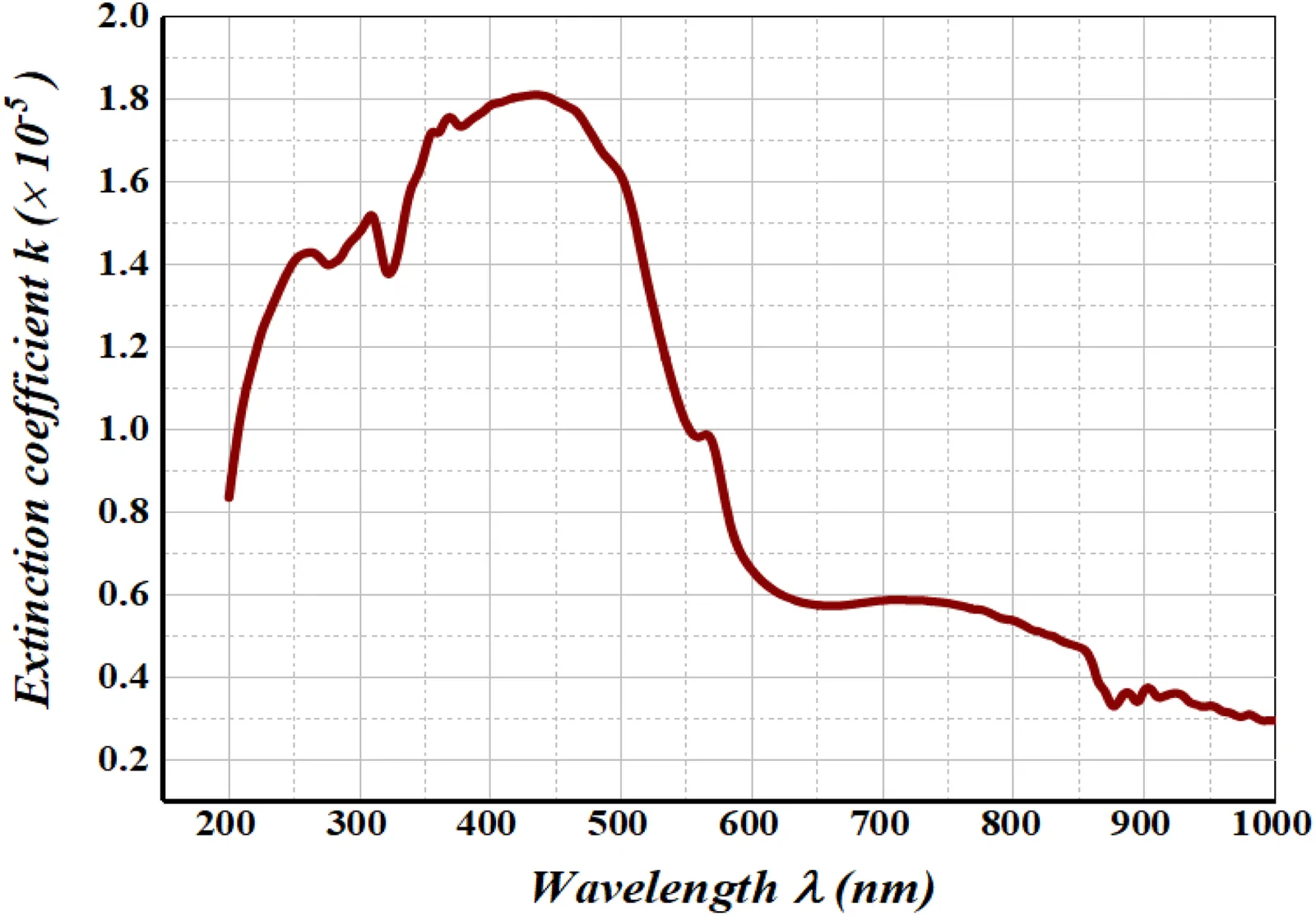
Dependence on wavelengh of the extinction coefficient (*k*) for the ZnMn_1.5_Al_0.5_O_4_ sample.

**Fig. 8:**
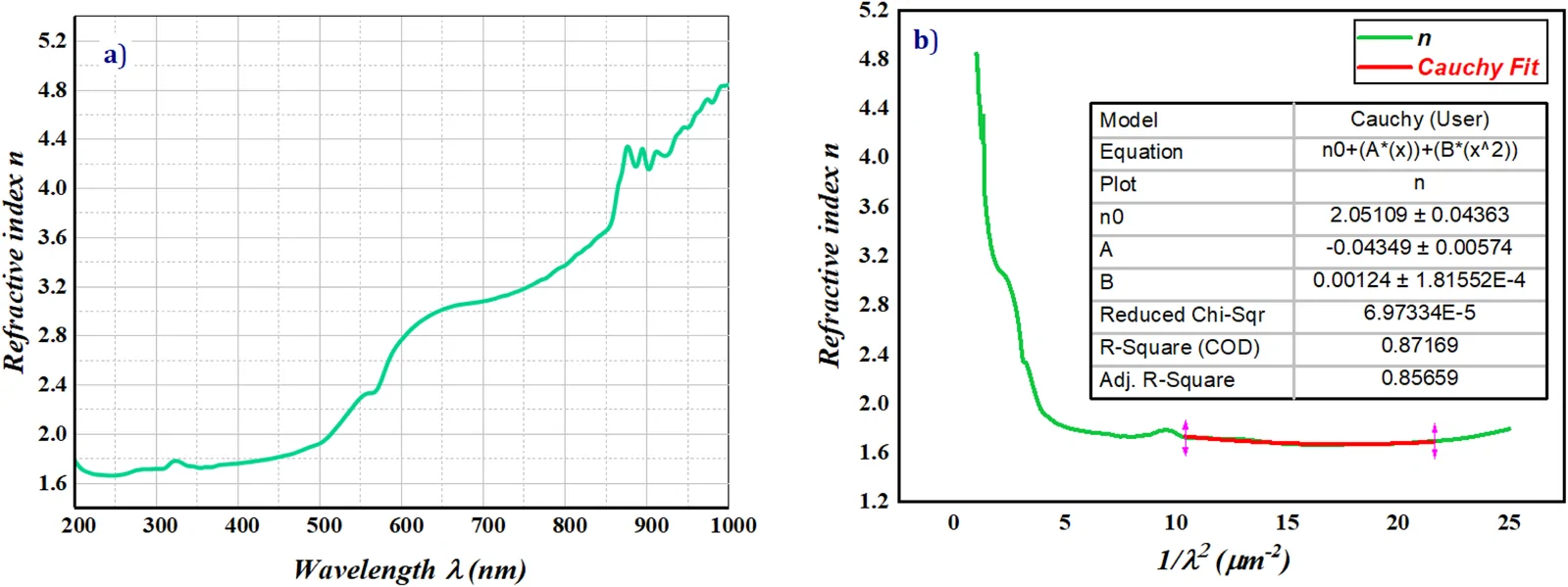
(a) Spectral evolution of refractive index *n vs.* wavelength, (b) theoretical adjustment of *n* by using the Cauchy model for the ZnMn_1.5_Al_0.5_O_4_ sample.

The spectral variations of the (*k*) and the (*n*) as a function of the incident radiation wavelength are presented in Fig. 7 and 8(a), respectively. Fig. 7 indicates that the extinction coefficient (*k*) exhibits very low values over the entire studied wavelength range, remaining far below unity. In the 190−445 nm wavelength range, *k* increases progressively and reaches higher values, indicating greater optical losses in this region. In contrast, within the 445−1000 nm range, *k* decreases significantly to its minimum value, highlighting a strong transmission of light. This suggests that the studied sample can be considered highly transparent in this spectral region.

Moreover, the spectral evolution of the *n* shows a gradual increase with increasing wavelength, indicating a reduction in the propagation speed of light within the material. The spectral behavior of the refractive index (*n*) can be described using the Cauchy dispersion function, expressed by the following equation:^67^25
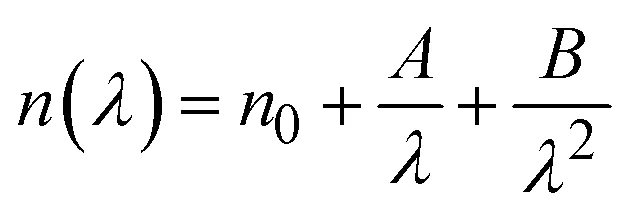
where the coefficients *n*_0_, *A* and *B* were determined by optimal fitting of the experimental data. The regression of *n* as a function of 
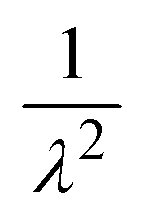
 is shown in Fig. 8(b), we have determined the values of the Cauchy constants: *n*_0_ = 2.0510 ± 0.0436, *A* = −0.0435 ± 0.0057 µm^−2^ and *B* = 0.0012 ± 0.0002 µm^−2^.

Based on the obtained value of the static refractive index, the reflection loss factor (*R*_L_) can be estimated using the following relation:^67^26
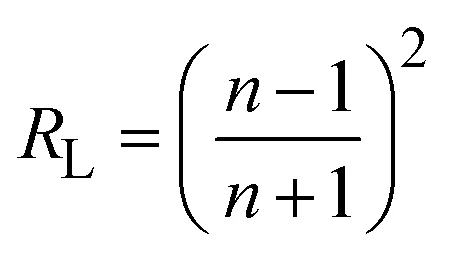


The *R*_L_ value of the studied manganese spinel ZMAO compound was estimated to be 0.118 (Table 2).

**Table 2:** Computed optical parameters of ZnMn_1.5_Al_0.5_O_4_ sample

Sample	ZnMn_1.5_Al_0.5_O_4_
*R*_L_	0.118
*R*_P_ (Å)	0.592
*R*_m_ (cm^3^ mol^−1^)	33.100
*α*_m_ (Å^3^)	13.100
*E*_0_ (eV)	5.150
*E*_d_ (eV)	11.360
*λ*_0_ (nm)	241
*S*_0_ (×10^−4^ nm^−2^)	1.445
*M*_(−1)_	2.210
*M*_(−3)_ (eV^−2^)	0.084
*f*_s_ (eV^2^)	58.500
*χ* ^1^	1.800
*χ* ^3^ (×10^−21^ m^2^ V^−2^)	2.264
*n*_2_ (×10^−20^ m^2^ V^−2^)	2.662

Furthermore, structural defects that may disrupt the periodicity of the electronic charge density can exist even in ideal crystal lattices.^68^ This phenomenon is mainly attributed to the interaction between charge carriers and lattice vibrations, leading to the formation of quasiparticles known as polarons.^69^

In particular, the presence of an excess charge induces a displacement of neighboring ions due to Coulomb interactions, thereby creating a local polarization cloud that follows the charge carrier as it moves through the crystal.^68^ Polaron formation is particularly favored in polar semiconductors and metal oxides due to strong electron–phonon coupling. It is even more likely at surfaces, where the crystal lattice is more flexible and atomic relaxations require less energy.^70^

The polaron radius (*R*_P_) can be calculated using the following expression:^70^27
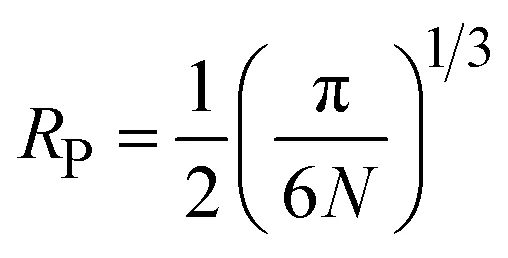


Herein, *N* represents the number of crystallographic sites per unit volume. Based on the tetragonally distorted spinel crystal structure (space group *I*4_1_/*amd*), which contains 32 octahedral and 64 tetrahedral interstitial sites per unit cell, the number of available cationic sites per unit volume *N* was calculated as 
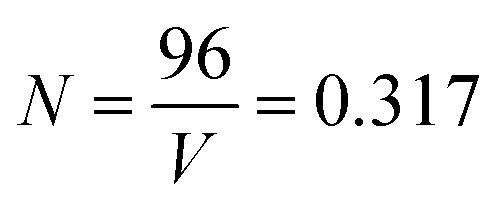
, where *V* denotes the unit-cell volume.

The molar refractivity, denoted as *R*_m_, of the studied material can be expressed as a function of the refractive index *n* and the molar volume *V*_m_, according to the Lorentz–Lorenz relation:^70^28
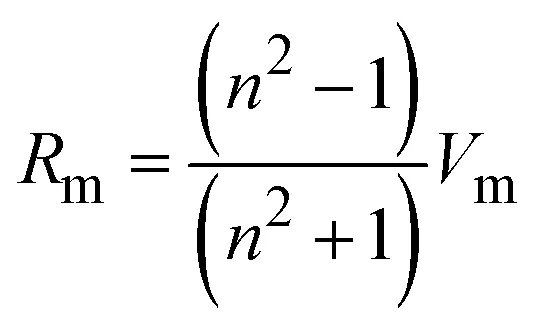


Accordingly, by assuming that *V*_m_ = *M*_w_/*ρ*_x-ray_, we obtain:29
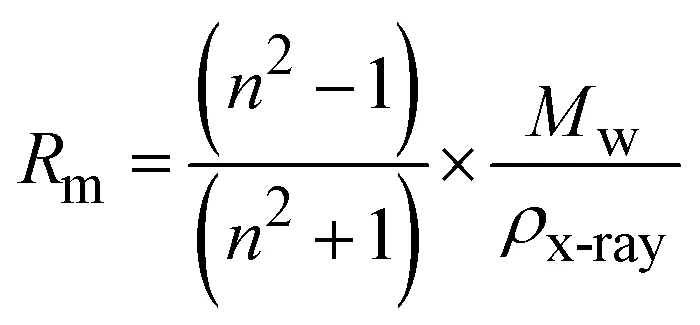


The term *M*_w_ represents the molar mass of the compound.

An additional parameter, known as the molar electronic polarizability *α*_m_, can be related to the molar refractivity *R*_m_ through the following expression:^70^30
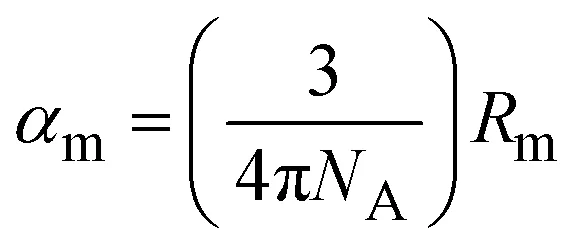
where *N*_A_ = 6.022 × 10^23^ mol^−1^ denotes Avogadro's number.

The calculated values of *R*_P_, *R*_m_ and *α*_m_ are summarized in Table 2.

It should be noted that the molar electronic polarizability *α*_m_ reflects the ability of a semiconductor to induce an electric dipole moment when subjected to an external electromagnetic field, responding to the incident field through the displacement of its charge carriers. Furthermore, this study demonstrates a direct proportionality between *R*_m_ and *α*_m_. This relationship is particularly useful for analyzing the nonlinear optical response, as it strongly depends on electronic polarizability.

To describe the dispersive behavior of the studied ZMAO material, it is appropriate to use the single effective oscillator model proposed by Wemple–DiDomenico.^71^ The dispersion of the refractive index (*n*) in semiconductors is a key parameter for the development of optical communication systems.^67^

In general, insulating and semiconducting materials exhibit an inverse relationship between the characteristic interband energy and the optical refractive index. This correlation, which is particularly valid for semiconductors, can also be related to the optical band-gap energy.^72^

The dispersion parameters are important for characterizing the optical dispersion behavior of materials and optimizing optical. In the Wemple–DiDomenico model, the single-oscillator energy (*E*_0_) and dispersion energy (*E*_d_) describe the electronic transition behavior and oscillator strength, respectively. These parameters are considered independent within the model.^40^ The moderate values of *E*_0_ = 5.15 eV and *E*_d_ = 11.36 eV are associated with a refractive index of *n* = 2.05, indicating moderate electronic polarizability and optical dispersion. Meanwhile, the very low extinction coefficient (*k* = 1.8 × 10^−5^) confirms negligible absorption in the investigated spectral region. Thus, variations in *E*_0_ and *E*_d_, reflecting changes in electronic transition energies and oscillator strength, are closely related to the dispersion of *n* and the absorption represented by *k*.^40^

Therefore, these two parameters can be determined as a function of the incident light frequency and the refractive index (*n*) using the following relations:^73^31
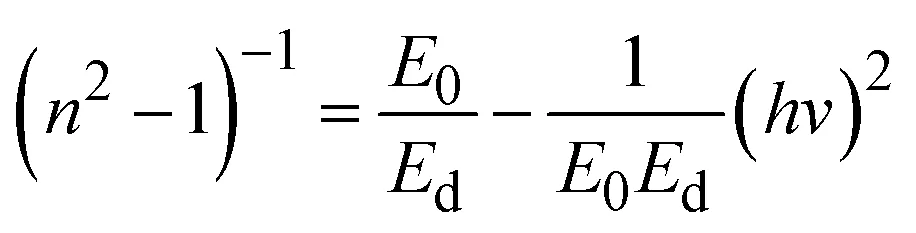
where *h* denotes the Planck constant (*h* = 6.62 × 10^−34^ J S).

**Fig. 9:**
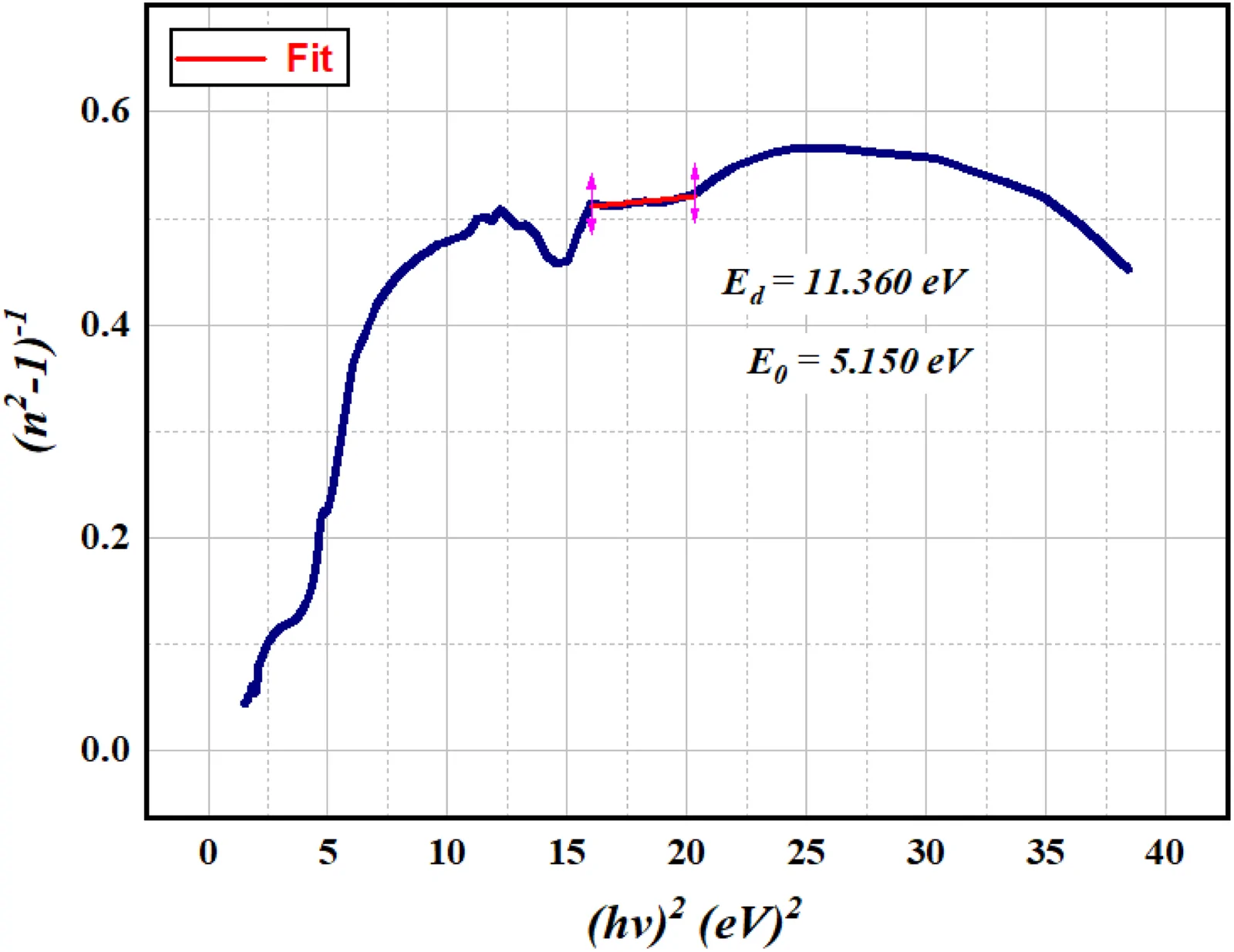
Linear fitting curves of (*n*^2^ − 1)^−1^ dependent on *hv* for the ZnMn_1.5_Al_0.5_O_4_ sample.

By fitting the linear region of the (*n*^2^ − 1)^−1^*versus* the square of the incident photon energy (*hv*), as illustrated in Fig. 9, the values of *E*_d_ and *E*_0_ can be extracted from the slope and the intercept, as summarized in Table 2.

The values obtained are generally consistent with those reported in the literature. Similarly, Sultan and Singh^74^ reported values for *E*_d_ (22.4–26.2 eV) and *E*_0_ (7.16–7.60 eV). Likewise, Zulqarnain *et al.* obtained values for *E*_d_ (2.61–30.49 eV) and *E*_0_ (8.44–10.25 eV).^75^ These results show that the dispersion parameters determined for ZMAO are within the same order of magnitude as those reported for spinel oxides.

Within the framework of the single-oscillator Sellmeier model, the oscillator strength (*S*_0_) can be determined. The parameter (*S*_0_) characterizes the strength of the electronic transitions responsible for the optical response of the material: a high value indicates strong optically active transitions and a stronger light–matter interaction, whereas a low value reflects a more limited contribution to the optical response. The wavelength (*λ*_0_) is related to the characteristic oscillator energy (*E*_0_) by the relation (*E*_0_ = *hc*/*λ*_0_). Thus, a low value of (*λ*_0_) corresponds to a high transition energy, whereas a large value indicates a lower energy.

**Fig. 10:**
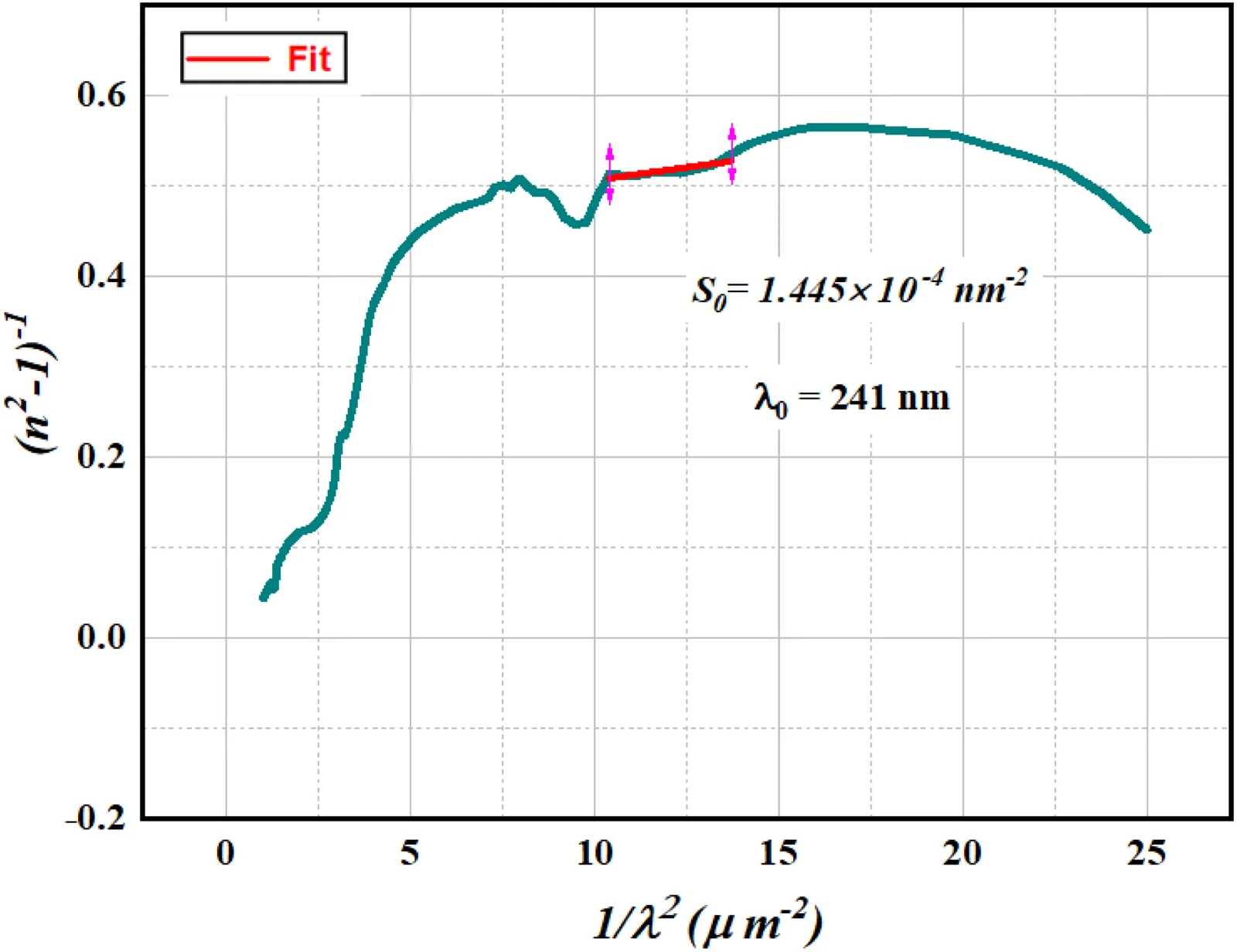
Linear fitting curves of (*n*^2^ − 1)^−1^ dependent on (1/*λ*)^2^ for the ZnMn_1.5_Al_0.5_O_4_ sample.

These parameters are related to the optical refractive index through the following expression:^73^32
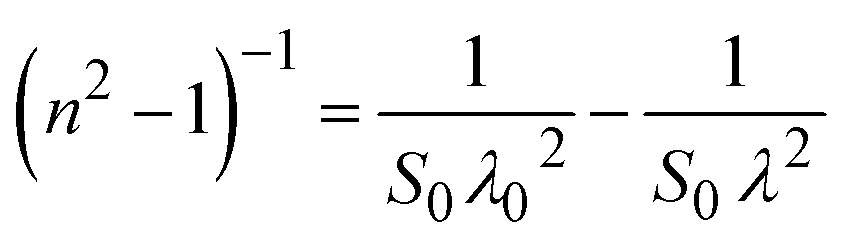
By fitting the linear region of the (*n*^2^ − 1)^−1^ variation *versus* the inverse square of the wavelength (1/*λ*^2^) (Fig. 10), the parameters *S*_0_ and *λ*_0_ are determined from the slope and *y*-intercept, respectively, as reported in Table 2. The obtained value of *λ*_0_ further confirms the validity of the 
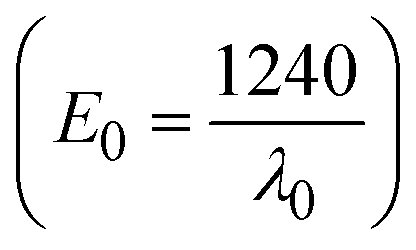
 parameter.

Furthermore, in the low-frequency region, the static refractive index (
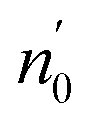
) and the zero-frequency dielectric constant can be estimated using the calculated values of *E*_0_ and *E*_d_:33
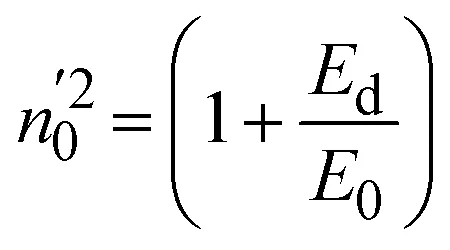


The 
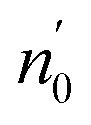
 obtained in the low-frequency region (
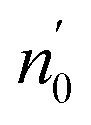
 = 1.790) shows good agreement with the value estimated from the theoretical Cauchy model (*n*_0_ = 2.051). This consistency confirms the reliability and accuracy of the different approaches employed in this study.

Based on the determined values of *E*_0_ and *E*_d_, the moments *M*_(−1)_ and *M*_(−3)_ were calculated using the following relations:^67^34
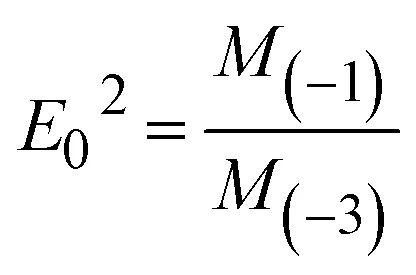
35
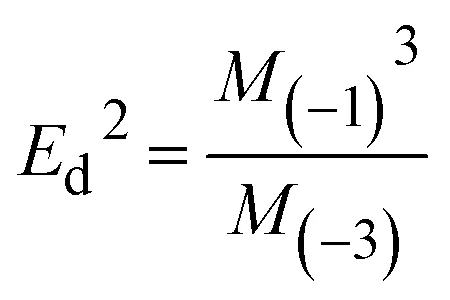


The oscillator strength (*f*_s_) is directly related to the dispersion parameters *E*_0_ and *E*_d_, can be expressed as follows:^59^

36 *f*_s_ = *E*_0_*E*_d_

The calculated values of *M*_(−1)_, *M*_(−3)_, and *f*_s_ for the ZMAO compound are summarized in Table 2.

Optical nonlinearity (ONL) is a rapidly growing research field due to its wide range of applications in advanced photonic devices. Nonlinear optics provides a deeper understanding of both elastic and inelastic light scattering phenomena occurring when intense electromagnetic radiation interacts with materials composed of atoms or molecules.^76^ This phenomenon arises when an external electric field *E⃑* induces a non-harmonic response of charge carriers within a nanostructured material. The induced electric dipoles, oscillating at the frequency of the applied field, add up to generate a macroscopic polarization in the irradiated medium.

Although higher-order contributions are usually weak due to small expansion coefficients, they can become significant when enhanced by resonance effects in the material.^76^

Therefore, the macroscopic polarizability can be expressed as a function of the electric field strength according to the following expression:^77^

37 *P* = *ε*_0_[χ^(1)^*E* + *χ*^(2)^*E*^2^ + *χ*^(3)^*E*^3^ + …]

where *ε*_0_ denotes the permittivity of free space (*ε*_0_ = 8.854 × 10^−12^ F m^−1^).

The linear optical susceptibility *χ*^(1)^ characterizes the response of the material to an electric field and describes the induced polarization proportional to this field. It is directly related to the linear refractive index and thus provides information about the strength of the light–matter interaction. A high value of *χ*^(1)^ indicates strong induced polarization and a significant linear optical response, whereas a low value reflects a more limited response. The susceptibility (*χ*^(2)^) is associated with phenomena such as second-harmonic generation and frequency mixing, whereas (*χ*^(3)^) is involved in nonlinear effects such as the optical Kerr effect, self-focusing, and self-phase modulation. These parameters therefore provide an assessment of the strength of the material's linear and nonlinear optical responses.

The linear susceptibility is directly related to the *n* value through the following expression:^78^38
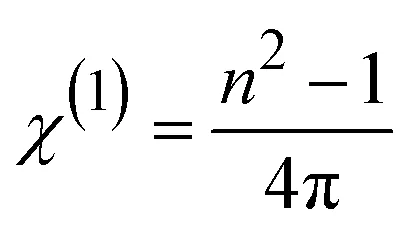


**Fig. 11:**
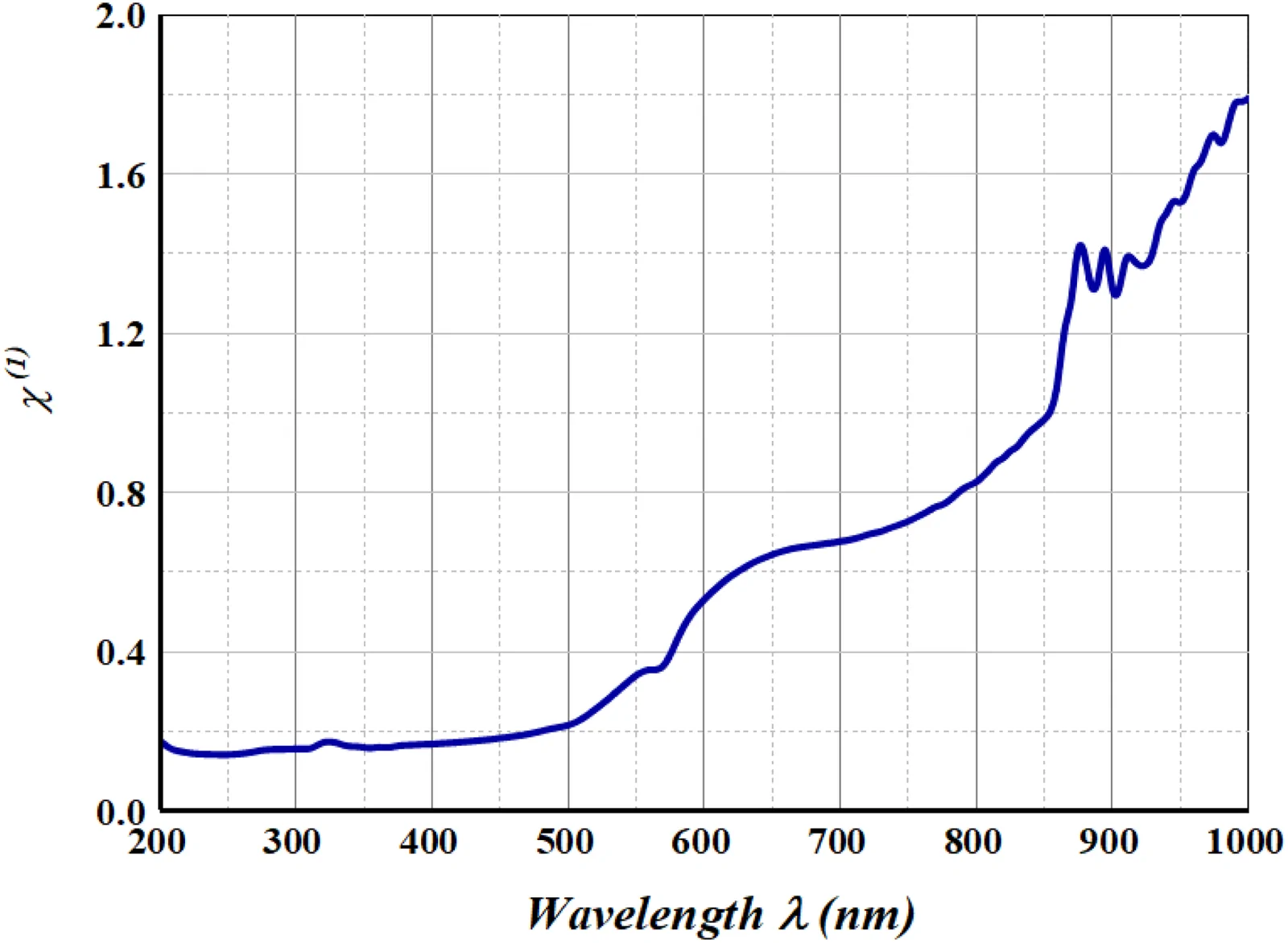
Spectral evolution of the first-order nonlinear optical susceptibility for the ZnMn_1.5_Al_0.5_O_4_ sample.

**Fig. 12:**
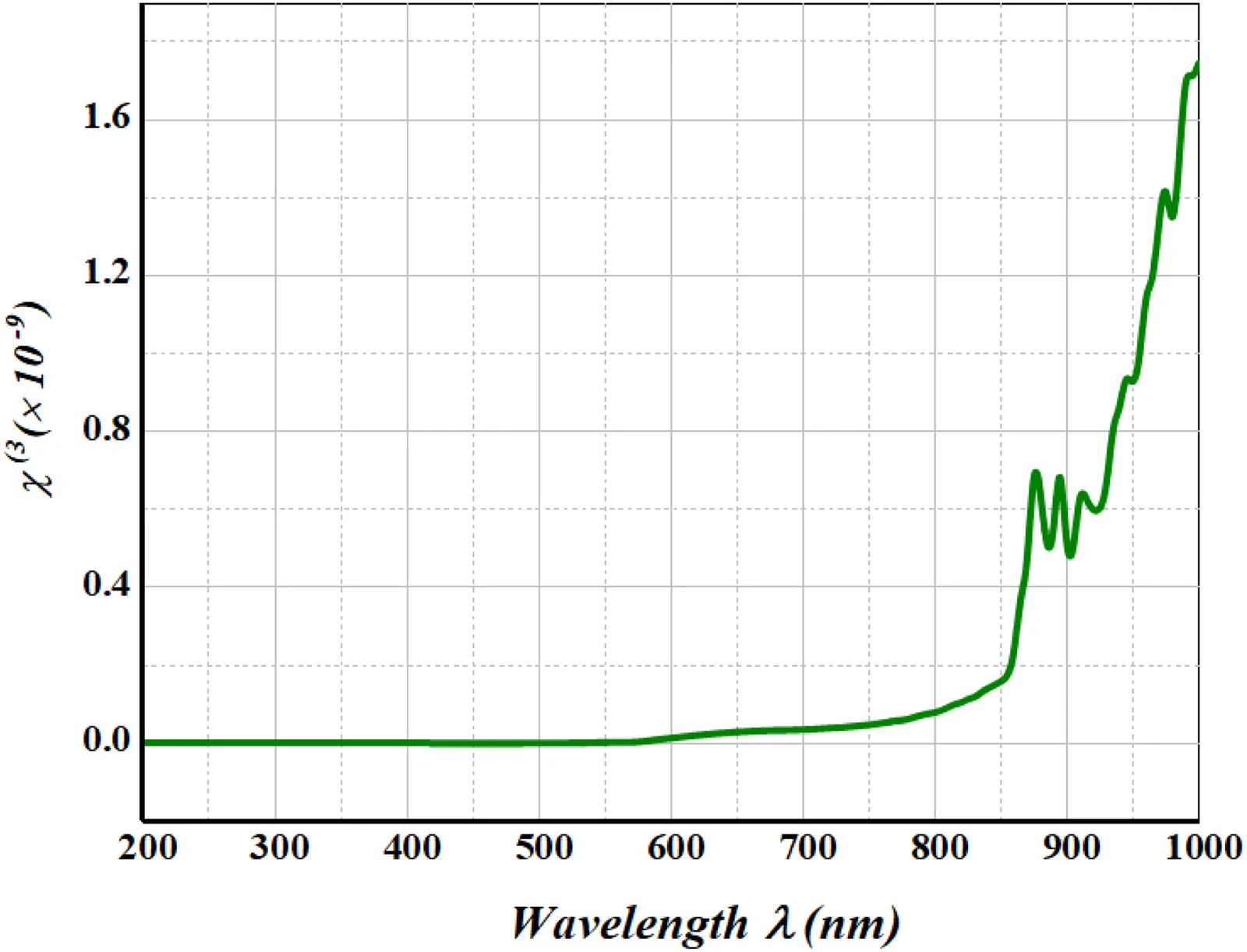
Spectral evolution of the third-order non-linear optical susceptibility for the ZnMn_1.5_Al_0.5_O_4_ sample.

The variation of the linear susceptibility as a function of the incident light wavelength is illustrated in Fig. 11. Based on this relationship, the *χ*^(3)^ can be estimated from the linear susceptibility as follows:^77^39
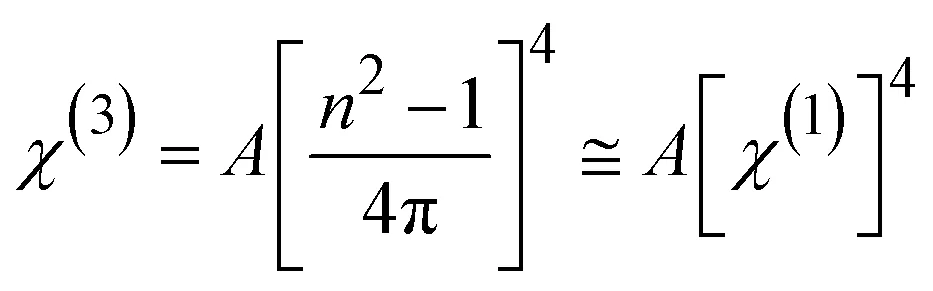
Here, *A* is a constant parameter estimated to be about 1.700 × 10^−10^ esu (where 1 esu corresponds to 1.400 × 10^−8^ m^2^ V^−2^). The dependence of the *χ*^(3)^ on the incident radiation wavelength is illustrated in Fig. 12. In the low-energy region, the *χ*^(3)^ can also be described in terms of *E*_0_ and *E*_d_ as follows:^59^40
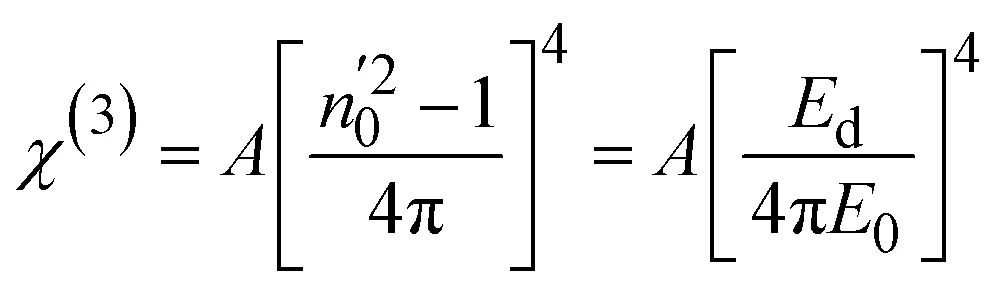


The nonlinear refractive index (*n*_2_) is related to the (
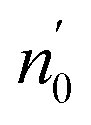
) and the χ^(3)^ through the following equation:^79^41
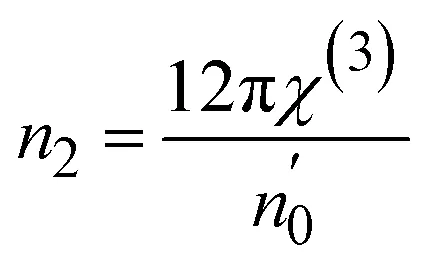


The calculated values of *χ*^(1)^, *χ*^(3)^, and *n*_2_ (Table 2) are in excellent agreement with those reported for other materials considered promising for nonlinear optical applications, especially in the fields of ultrafast optical communication and optical information processing. A comparison of the nonlinear optical parameters shows that the investigated ZMAO sample exhibits a linear susceptibility, *χ*^(1)^ = 1.800, which is significantly higher than those reported for BaTi_1−*x*_Fe_*x*_O_3_ (0.192–0.262),^80^ Sr_3−*x*_Pb_*x*_Fe_2_TeO_9_ (0.129–0.223),^86^ and Cd_1−*x*_Eu_*x*_O (0.270–0.445).^83^

In contrast, its third-order nonlinear susceptibility, χ^(3)^ = 2.264 × 10^−21^ m^2^ V^−2^, is comparable to those reported for BaTiO_3_ (3.249 × 10^−21^)^80^ and Sr_1.5_Pb_1.5_Fe_2_TeO_9_ (2.229 × 10^−21^)^81^ but remains lower than the high values reported for Cd_1−*x*_Eu_*x*_ (9.240 × 10^−20^–2.520 × 10^−20^).^83^ Similarly, the nonlinear refractive–index coefficient *n*_2_ = 2.662 × 10^−20^ m^2^ W^−1^ for ZMAO is lower than those reported for BaTi_1−*x*_Fe_*x*_O_3_ (2.687 × 10^−19^–7.495 × 10^−19^)^80^ and Cd–Eu, while remaining of the same order of magnitude as that reported for Sr_1.5_Pb_1.5_Fe_2_TeO_9_ (6.948 × 10^−20^).^81^ These results indicate that ZMAO is distinguished primarily by its particularly high *χ*^(1)^ value, whereas its third-order nonlinear optical susceptibility, *χ*^(3)^, and nonlinear refractive index, *n*_2_, are moderate compared with those of certain reference materials. Nevertheless, the combination of a high linear optical response and measurable third-order nonlinear optical parameters suggests that ZMAO may have potential for optical and photonic applications.^84^

#### 3.3.5 Optical conductivity behavior

The optical conductivity (*σ*_opt_) and the electrical conductivity (*σ*_ele_) provide complementary information about the electronic response of the material. Optical conductivity characterizes the response of electrons to the incident electromagnetic field and provides information particularly about electronic transitions and photoexcitation processes.^82^ A high value of *σ*_opt_ indicates stronger light–matter interaction and a more pronounced electronic response to radiation. In contrast, electrical conductivity describes the transport of charge carriers under an applied electric field and mainly depends on their concentration and mobility. A high value of *σ*_ele_ therefore indicates more efficient charge transport. The analysis of these two quantities thus makes it possible to evaluate the electronic response to optical excitation and the electrical transport properties of the material, respectively.

Based on the optical absorption coefficient, these two parameters can be expressed as follows:^85^42
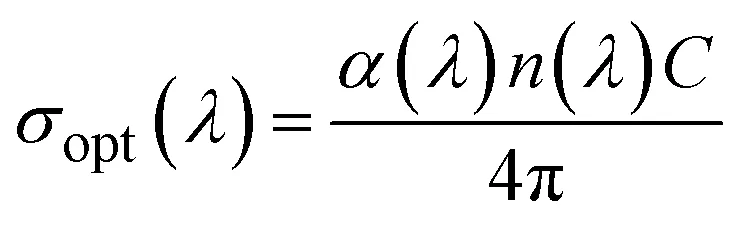
43
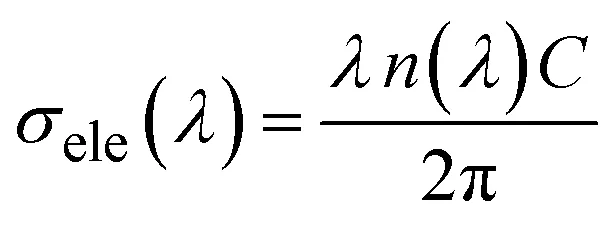
where *C* is the speed of light in vacuum, approximately 3 × 10^8^ m s^−1^.

**Fig. 13:**
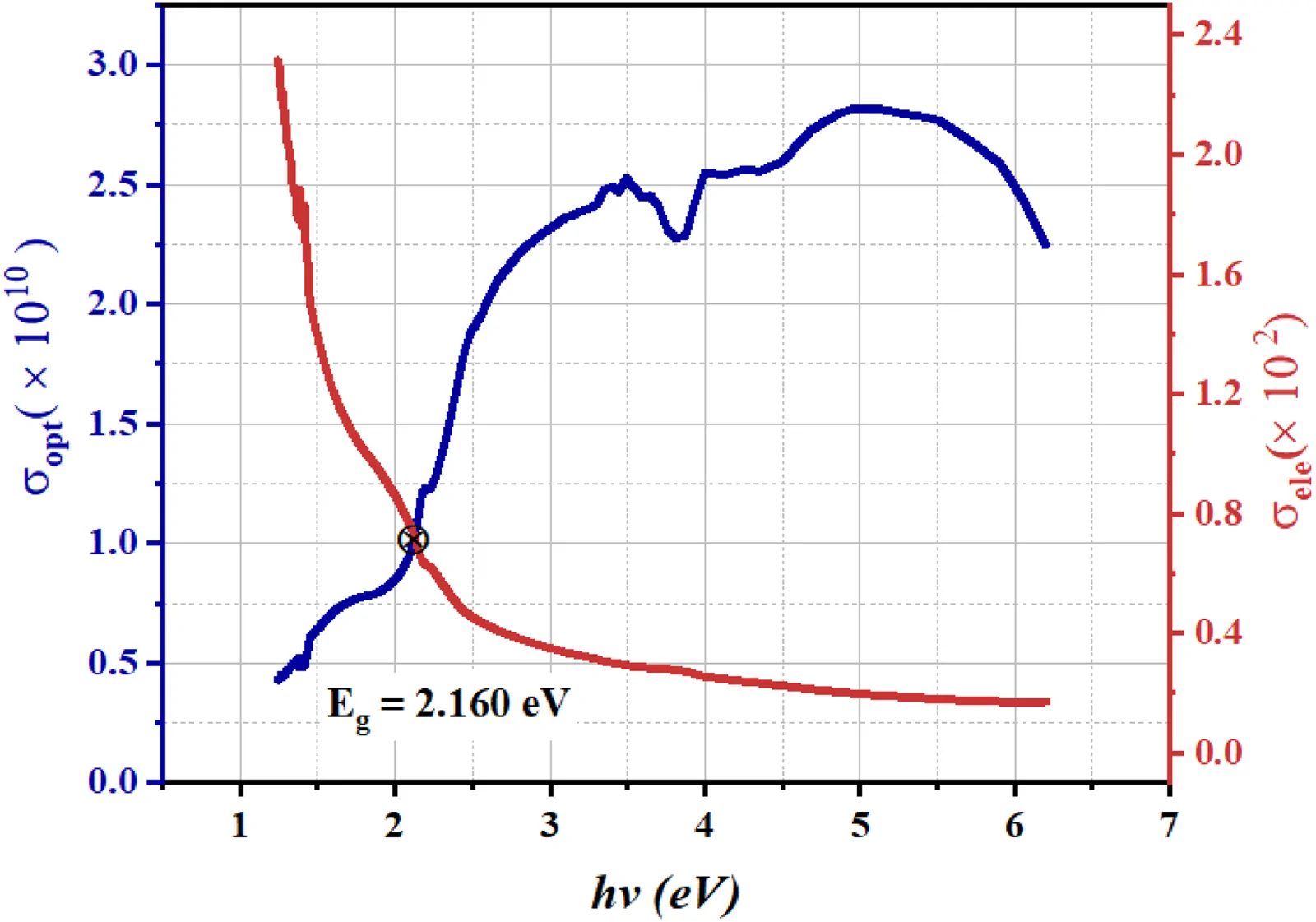
Dependence of the optical (*σ*_opt_) and the electrical conductivity (*σ*_ele_) on *hν* for the ZnMn_1.5_Al_0.5_O_4_ sample.

Fig. 13 illustrates the variation of the *σ*_opt_ and *σ*_ele_ as a function of the *hv* for the ZnMn_1.5_Al_0.5_O_4_ compound. A marked increase in *σ*_opt_ is observed in the short-wavelength region, indicating strong light absorption and efficient charge-carrier excitation under high-energy irradiation.

Furthermore, the intersection points between the *σ*_opt_ and *σ*_ele_ curves enable the estimation of the optical band gap *E*_g_, which is in good agreement with the value obtained from the Tauc analysis. The high optical conductivity, reaching approximately 2.8 × 10^10^, indicates a strong response of ZnMn_1.5_Al_0.5_O_4_ to electromagnetic radiation and reflects the significant contribution of electronic transitions to its optical response, highlighting its potential applications.^86^ In contrast, the relatively lower values of *σ*_ele_ compared with *σ*_opt_, particularly at high photon energies, indicate less efficient charge-carrier transport. This behavior can be attributed to the presence of potential barriers and localized states that hinder carrier motion, as some electrons may not possess sufficient energy to overcome these barriers and contribute effectively to electrical conduction.^87^ Thus, the combined analysis of *σ*_opt_ and *σ*_ele_ provides complementary information on the electronic behavior of ZnMn_1.5_Al_0.5_O_4_, highlighting both its pronounced optical response and the limitations governing charge transport.

In this study, the optical properties of ZnMn_1.5_Al_0.5_O_4_, were determined from UV-Visible measurements. The reflectance spectra were converted using the Kubelka–Munk function to evaluate the absorption coefficient. This coefficient was used to determine an optical band gap energy of *E*_g_ = 2.160 ± 0.017 eV using the Tauc method, as well as an Urbach energy of *E*_U_ = 0.376 ± 0.006 eV. The refractive index and calculated extinction coefficient were found to be *n* = 4.8 and *k* = 1.8 × 10^−5^, respectively.

The analysis of optical dispersion yielded the parameters *E*_0_ = 5.150 eV and *E*_d_ = 11.360 eV, together with a characteristic oscillator wavelength of *λ*_0_ = 241 nm and an oscillator strength of *S*_0_ = 1.445 × 10^−4^ nm^−2^. These parameters provide information on the electronic transitions responsible for the dispersive behavior of the material.

The estimated nonlinear optical parameters are *χ*^(1)^ = 1.800, *χ*^(3)^ = 2.264 × 10^−21^ m^2^ V^−2^, and *n*_2_ = 2.662 × 10^−20^ m^2^ V^−2^, indicating a notable nonlinear optical response. The optical (2.8 × 10^10^) and electrical (3 × 10^2^) conductivities complement this characterization by providing additional information on the electronic response and charge-carrier transport.

Thus, these parameters establish a correlation between the UV-Visible measurements and the derived optical properties, providing a comprehensive understanding of the electronic structure, optical transitions, dispersion, charge transport, and linear and nonlinear optical responses of ZMAO.

#### 3.3.6 Optical dielectric constant

The optical dielectric constant is a complex quantity composed of two parts: the real part (*ε*_r_), which describes the refraction of light as it propagates through the material, and the imaginary part (*ε*_im_), which characterizes the absorption and attenuation of incident waves.

Both components can be expressed in terms of the refractive index (*ε*_r_) and the extinction coefficient (*ε*_im_) as follows:^85^

44 *ε*_r_(*λ*) = *n*^2^(*λ*) − *k*^2^(*λ*)

45 *ε*_im_(*λ*) = 2*n*(*λ*)*k*(*λ*)

**Fig. 14:**
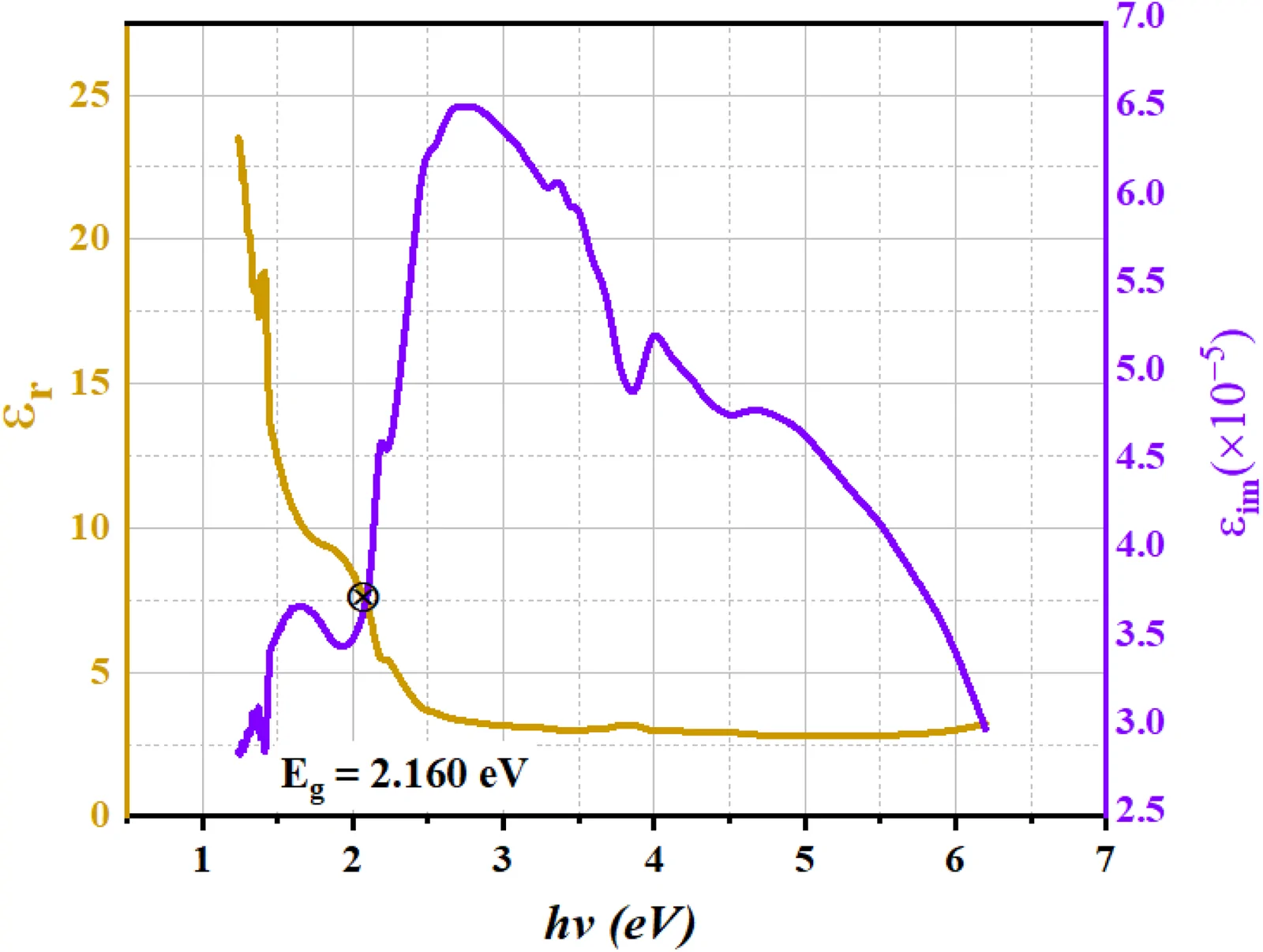
Dependence of *ε*_r_ and *ε*_im_ on *hν* for the ZnMn_1.5_Al_0.5_O_4_ sample.

According to the spectral evolution of the *ε*_r_ and *ε*_im_ as a function of (*hν*) (Fig. 14), the *ε*_r_, exhibits a trend similar to that of the refractive index (*n*), which in turn follows a behavior comparable to the reflectance spectrum. In contrast, *ε*_im_ coefficient shows a behavior analogous to that of the extinction coefficient (*k*), which is strongly influenced by the optical absorption of the material. Moreover, the intersection points between the *ε*_r_, and *ε*_im_ allow the estimation of the band gap energy (*E*_g_ = 2.160 ± 0.017 eV), in good agreement with the previously obtained results.

Furthermore, the dielectric loss, also known as the dissipation factor tan(*δ*), represents the portion of electrical energy dissipated as heat within semiconducting materials. It is defined as the ratio between the *ε*_r_ and *ε*_im_:^88^46
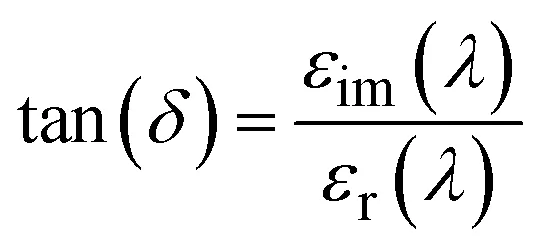


**Fig. 15:**
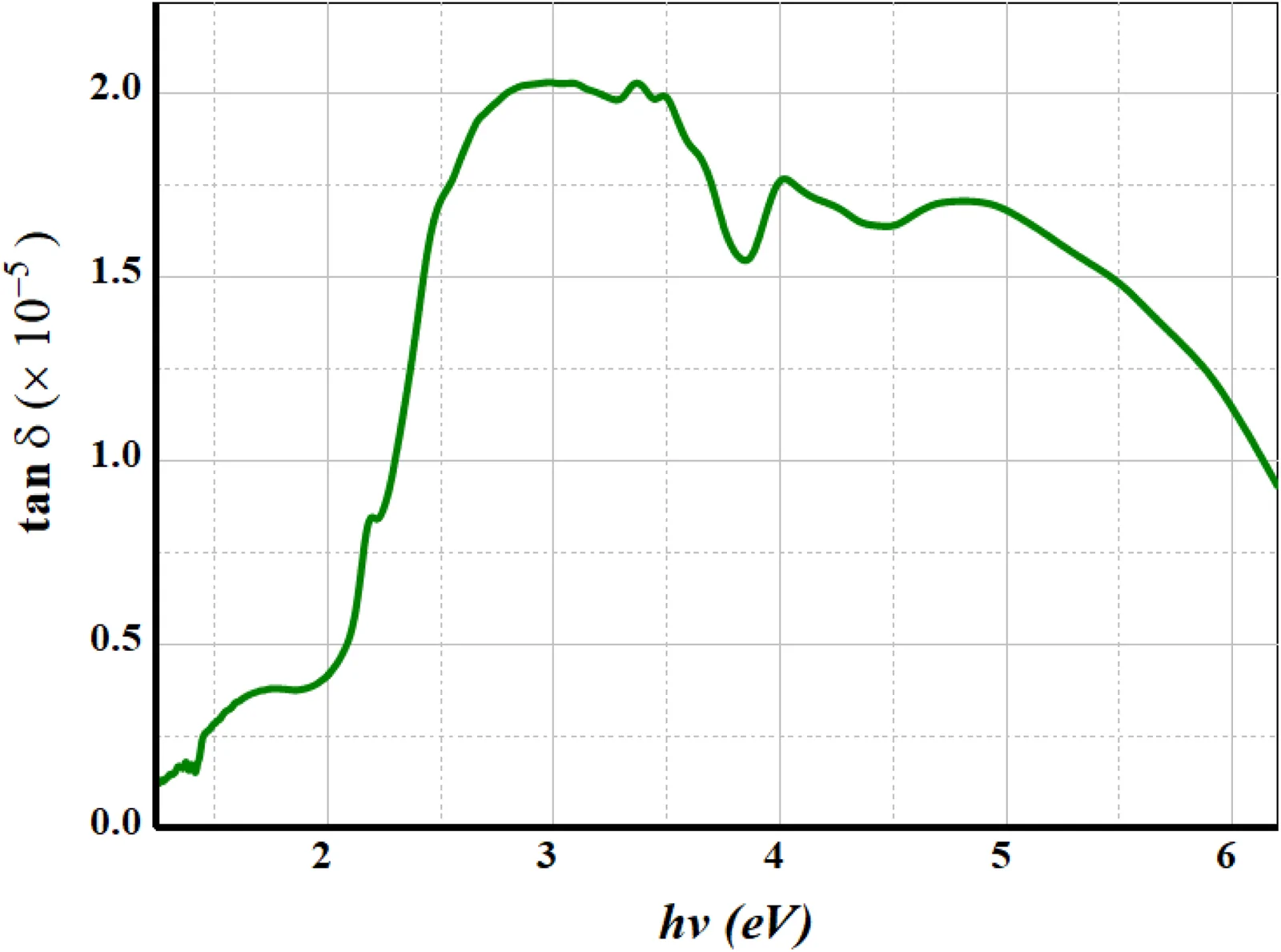
Dependence of tan *δ* on *hν* for the ZnMn_1.5_Al_0.5_O_4_ sample.

The variation of the tan(*δ*) as a function of *hv* is shown in Fig. 15. The spectral behavior of tan(*δ*) exhibits generally low values, indicating a weak dissipation of the light energy as it propagates through the studied material. Moreover, Fig. 15 shows a noticeable increase in the energy range between 1.250 and 2.750 eV, suggesting the presence of activation processes within this spectral region.

#### 3.3.7 Optical modulus

The optical modulus is an important physical quantity used to describe the optical properties of materials. It is particularly useful for identifying the allowed interband optical transitions within a material. The complex optical modulus (M*) is related to the complex dielectric function (ε*) by the following relation:^89^47
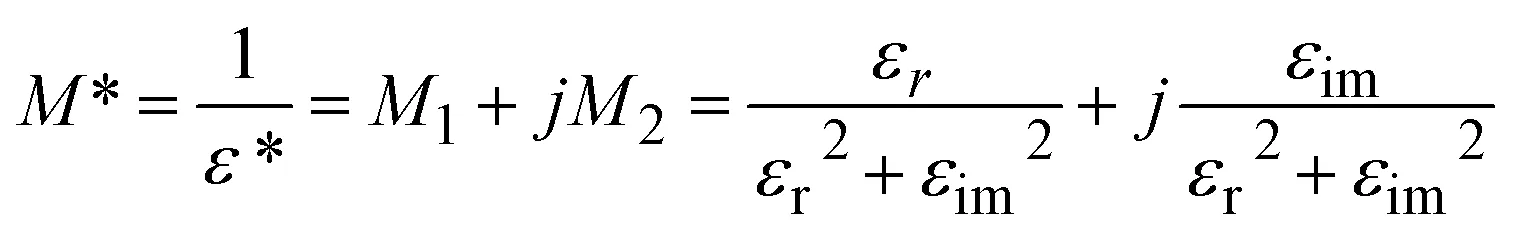


**Fig. 16:**
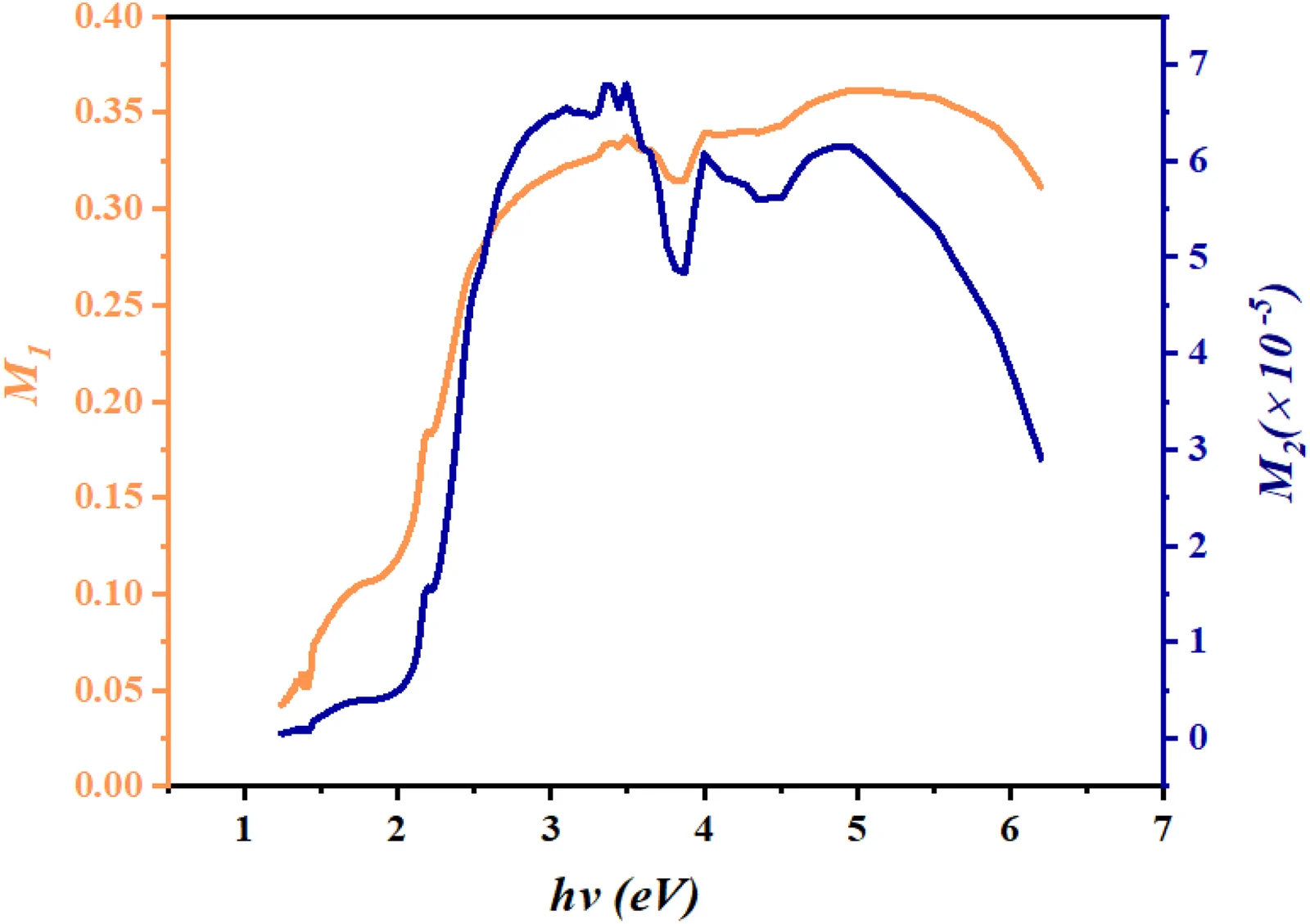
Dependence of *M*_1_ and *M*_2_ on *hν* for the ZnMn_1.5_Al_0.5_O_4_ sample.

Fig. 16 presents the real (*M*_1_) and imaginary (*M*_2_) parts of the optical (complex electric) modulus as a function of (*hν*). The complex modulus is a useful parameter for analyzing relaxation mechanisms in materials. *M*_1_ increases with *hν*, reaches a plateau between 3 and 5 eV, and then slightly decreases at higher energy, indicating polarization saturation and stabilization of the electrical response. In contrast, *M*_2_ rises rapidly to a maximum around 3 eV, reflecting a dominant relaxation process, then shows fluctuations due to the coexistence of multiple relaxation mechanisms before gradually decreasing at higher energies. Overall, this behavior suggests reduced dissipative losses and a transition toward a more stable regime, highlighting dipolar relaxation and localized charge transport in the studied material.^90^

## 4 Photoluminescence study

**Fig. 17:**
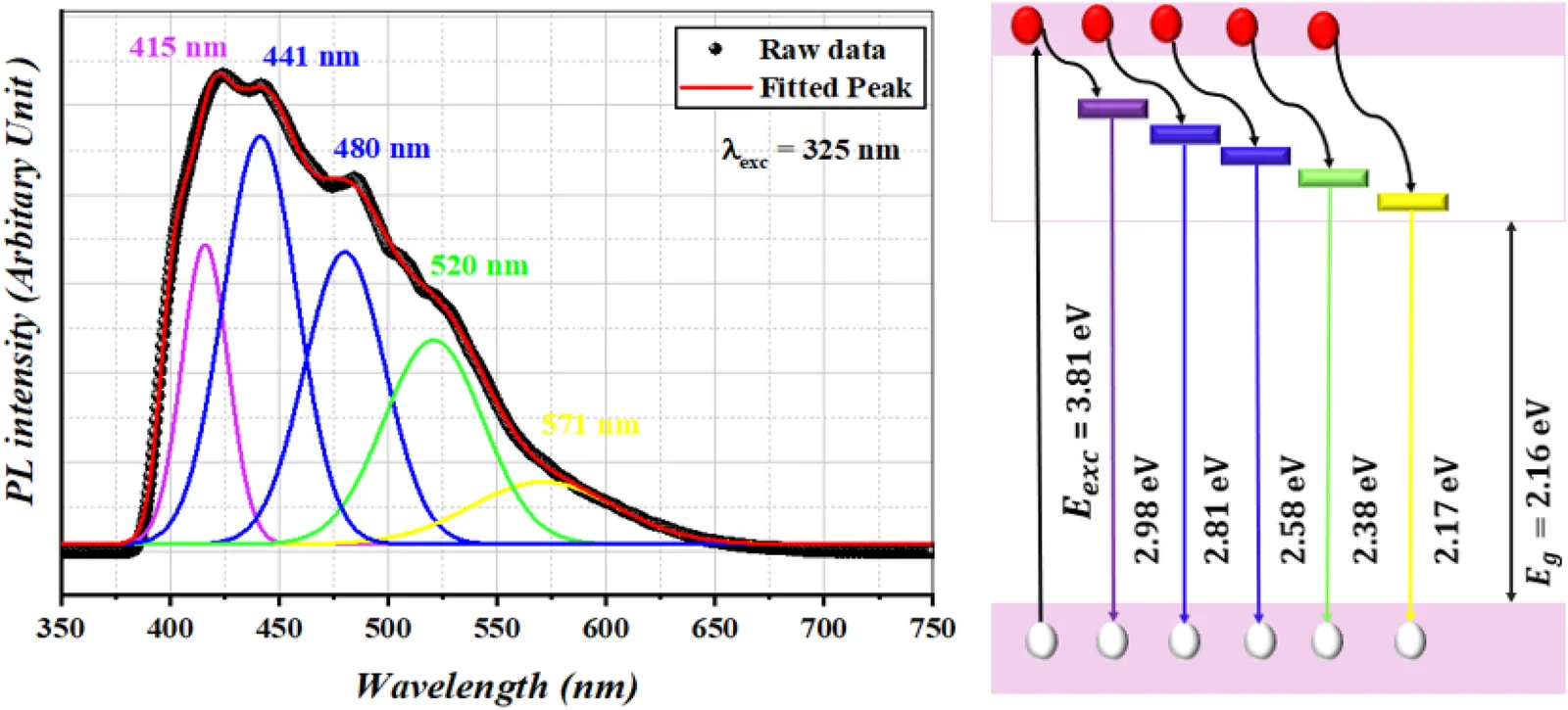
Experimental PL spectrum of ZnMn_1.5_Al_0.5_O_4_ measured under *λ*_exc_ = 325 nm excitation, along with the Gaussian fitting results and the corresponding electronic transition model explaining the observed luminescence emissions.

The photoluminescence (PL) spectroscopy is a powerful technique for investigating the luminescence characteristics of semiconductor materials and gaining insight into charge-carrier recombination processes. Fig. 17 presents the room-temperature PL spectrum of ZnMn_1.5_Al_0.5_O_4_ recorded under an excitation wavelength of *λ*_exc_ = 325 nm, and Gaussian deconvolution (the black curve depicts the experimental spectrum; the red curve represents the overall fitting obtained from Gaussian deconvolution). In this Figure, the experimental spectrum is depicted by the black curve, while the red curve represents the overall fitting obtained from Gaussian deconvolution The colored sub-peaks correspond to the individual emission bands resolved through the deconvolution procedure, accompanied by the corresponding electronic transition model.

The room-temperature PL spectrum was deconvoluted into five emission components centered at approximately 415, 441, 480, 520, and 571 nm, corresponding to photon energies of 2.98, 2.81, 2.58, 2.38, and 2.17 eV, respectively. Considering the experimentally determined optical band gap of approximately 2.160 ± 0.017 eV, the emission centered at 571 nm can be reasonably attributed to near-band-edge radiative recombination. In contrast, the shorter-wavelength components observed at 415–520 nm indicate the involvement of additional radiative pathways associated with localized electronic states, Mn-related transitions, and structural or defect states. In particular, the green emission near 520 nm has been reported in ZnMn_2_O_4_-based systems and associated with defect-related luminescence.^91^ The incorporation of Al^3+^ may additionally modify the local cationic environment and structural disorder, thereby affecting these radiative recombination pathways.

**Fig. 18:**
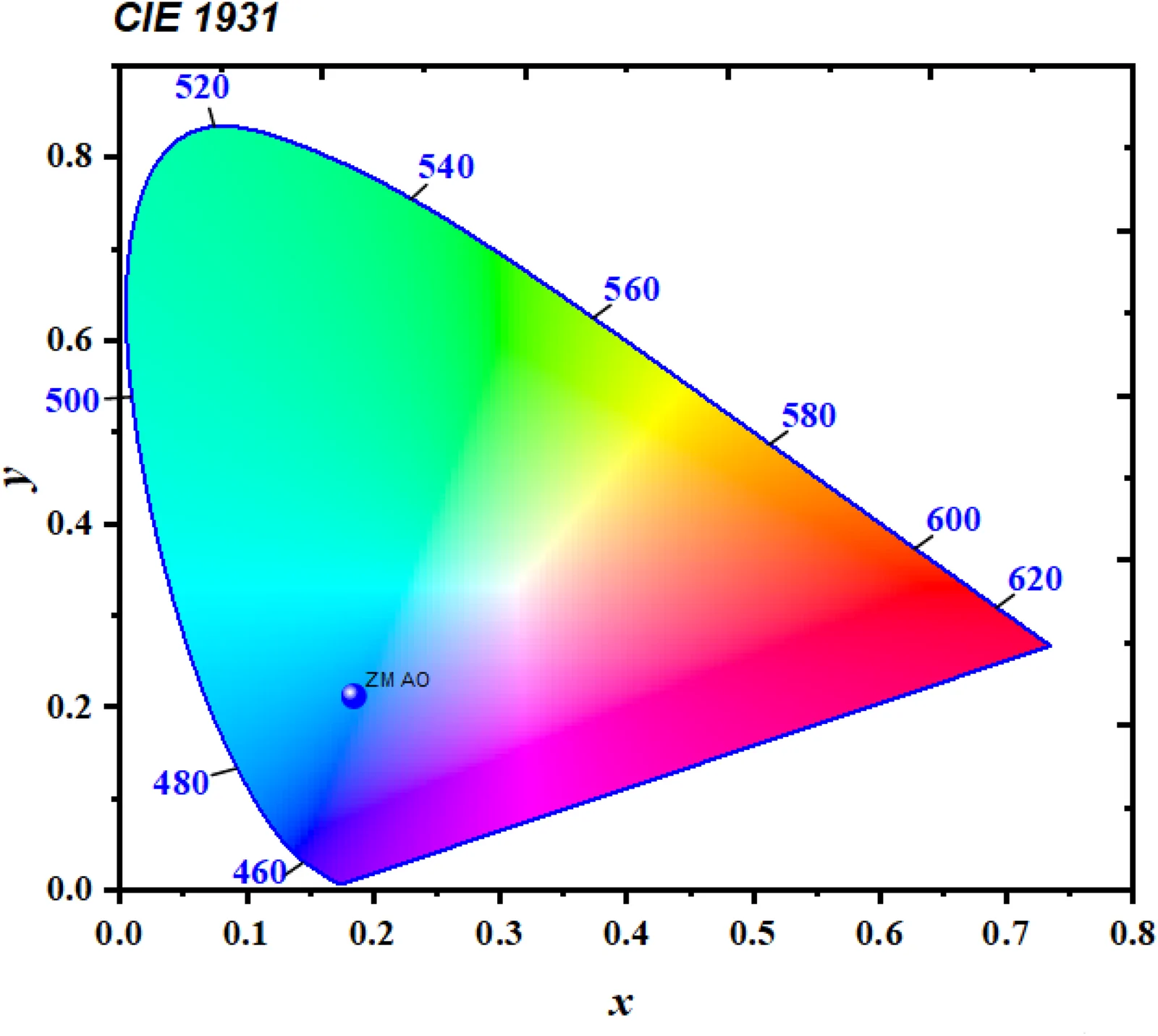
CIE 1931 chromaticity diagram of the ZnMn_1.5_Al_0.5_O_4_, excited at *λ*_ex_ = 325 nm.

To gain further insight into the emission properties of ZnMn_1.5_Al_0.5_O_4_, CIE 1931 chromaticity coordinates (*x* ≈ 0.18, *y* ≈ 0.21) were extracted from the photoluminescence spectrum (Fig. 18). These values locate the emission in the blue–cyan region, indicating a dominant blue-green luminescence with high color purity, as evidenced by its significant distance from the white-light region. This behavior is attributed to Mn-related electronic transitions and defect states introduced by partial Al^3+^ substitution, which modifies the local crystal field and influences radiative recombination pathways. Overall, these results highlight the potential of ZnMn_1.5_Al_0.5_O_4_ for device applications, particularly in phosphors for LEDs and solid-state lighting.

## 5 Conclusion

In this work, the ZMAO compound was successfully synthesized by the sol–gel auto-combustion method. Rietveld refinement confirmed the formation of a tetragonal spinel structure with the *I*4_1_/*amd* space group. Morphological analysis revealed the formation of relatively homogeneous and well-distributed particles. Optical investigations showed that ZMAO exhibits semiconductor behavior characterized by a direct optical band gap of *E*_g_ = 2.160 ± 0.017 eV. This relatively low value favors absorption in the visible region and highlights the effect of the partial substitution of Mn^3+^/Mn^4+^ ions by Al^3+^ ions. Furthermore, the Urbach energy, estimated at *E*_U_ = 0.376 ± 0.006 eV, indicates the presence of localized electronic states associated with defects and structural disorder.

The optical dispersion study using the Wemple–DiDomenico model allowed the determination of the dispersion parameters *E*_0_ = 5.150 eV and *E*_d_ = 11.360 eV. These parameters provide complementary information on the electronic transitions and confirm the dispersive behavior of the material. ZMAO also exhibits a refractive index of *n*_0_ ≈ 2.05, together with an extremely low extinction coefficient, *k* = 1.8 × 10^−5^. The combination of a relatively high refractive index and a very low extinction coefficient indicates low absorption in certain regions of the spectral range and highlights the potential of the material for optical applications. In addition, the low dielectric losses observed at high frequencies indicate a relatively stable electrical response, further supporting the potential of ZMAO for electronic and functional devices. The investigation of the nonlinear optical properties also revealed a significant optical response of ZMAO. The third-order nonlinear optical susceptibility reaches *χ*^(3)^ = 2.264 × 10^−21^ m^2^·V^−2^, while the nonlinear refractive index is estimated at *n*_2_ = 2.662 × 10^−20^ m^2^·V^−2^. These values indicate a significant nonlinear interaction between the material and the incident electromagnetic field and highlight the potential of ZMAO for futur nonlinear optical and photonic applications. Photoluminescence measurements revealed several emission bands in the visible region, indicating the presence of different radiative recombination centers as well as electronic transitions involving defect-related states. These emissions reflect the complexity of the electronic structure of ZMAO and the influence of Al^3+^ ion substitution on electron–hole recombination mechanisms. Furthermore, the chromaticity analysis in the CIE color space confirmed the optical potential of ZMAO by revealing emission characteristics compatible with applications in light-emitting diodes (LEDs) and semiconductor-based lighting systems.

These results provide a solid basis for future studies aimed at optimizing the performance of ZMAO through precise control of doping, composition, particle size, morphology, or surface modification, in order to further enhance its performance for next-generation energy technologies.

## Conflicts of interest

There are no conflicts to declare.

## Data Availability

The data supporting this study are available upon request but not for sharing.
